# RelA Inhibits Embryonic Myogenesis by Coordinately Regulating a Novel Distal Enhancer of Myogenin

**DOI:** 10.1002/advs.202503712

**Published:** 2025-07-28

**Authors:** Md Nazmul Hossain, Yao Gao, Sharmeen Islam, Li‐Wei Chen, Xinrui Li, Zhongyun Kou, Nathan C Law, Jeanene Marie de Avila, Mei‐Jun Zhu, Min Du

**Affiliations:** ^1^ Nutrigenomics and Growth Biology Laboratory Pullman WA 99164 USA; ^2^ Department of Animal Sciences Washington State University Pullman WA 99164 USA; ^3^ School of Food Science Washington State University Pullman WA 99164 USA

**Keywords:** embryonic myogenesis, enhancer, inflammation, maternal obesity, myogenin, RelA

## Abstract

Skeletal muscle is crucial for lifelong metabolic health, with its development initiating during embryogenesis and guiding later growth. Single‐cell RNA sequencing of E13.5 mouse embryos suggests that maternal obesity impairs embryonic myogenesis, primarily during myotube formation, and is correlated with the downregulation of myogenin (*Myog)* expression. Spatial transcriptomic sequencing further confirms these findings. Conversely, maternal obesity induces the upregulation of RelA, a transcription factor and inflammatory signaling effector, in the myogenic cells of obese embryos. Additionally, single‐cell ATAC sequencing suggests that a cis‐regulatory region, located 4 kb proximal to the *Myog* promoter, exhibits coordinated expression with *Myog* and acts as an enhancer of *Myog*. In obese embryonic myogenic cells, RelA is recruited to this region, inhibiting *Myog* expression and myotube formation. Notably, in vitro RelA knockdown or inhibition rescues *Myog* expression and promotes myogenic differentiation. Similarly, metformin treatment of obese mothers restores embryonic *Myog* expression and myogenesis by suppressing RelA. Together, the multi‐omics analyses demonstrate that RelA targets a distal *Myog* enhancer to coordinately inhibit *Myog* expression and embryonic myogenesis.

## Graphical abstract

Maternal obesity (MO) accounts for more than 40% of pregnancies in the United States, induces inflammation, and impairs embryonic myogenesis, known to program muscle dysfunction in offspring. Inflammation enhances the binding of RelA to a novel distal enhancer located 4 kb upstream of *myogenin* promoter to inhibit embryonic myogenesis, which is recovered by metformin treatment during early pregnancy.

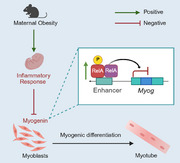

## 1 Introduction

Skeletal muscle is the major tissue for locomotion and metabolism, constituting 30–40% of body mass.^[^ ^1^, ^2^, ^3^ ^]^ Myogenesis starts during early embryogenesis, and the majority of the primary and secondary muscle fibers form during the prenatal stage through a sequential progression of myogenic progenitor cells to multinucleated myotubes and fibers. During the early stage of embryogenesis, myogenic progenitor cells originate from the dermomyotome of embryonic mesoderm and migrate to their designated locations in the body to develop into various muscle groups.^[^ ^4^ ^]^ Embryonic myogenesis starts around embryonic day 9.5 (E9.5) in the mesoderm of mice when PAX3 and PAX7‐positive myogenic progenitor cells first appear.^[^ ^5^, ^6^ ^]^ During E10.5 to E12.5, these progenitor cells express the first myogenic regulatory factor (MRF), MYF5, which initiates myogenic lineage commitment.^[^ ^7^ ^]^ A portion of MYF5+ cells also contributes to the brown adipocyte lineage.^[^ ^8^ ^]^ Subsequently, MYOD1, another member of MRFs, appears and enforces the differentiation of myogenic progenitor cells to myoblasts.^[^ ^9^ ^]^ Later, myoblasts undergo terminal differentiation and fuse to form multinucleated myotubes, which is governed by myogenin (*Myog*).^[^ ^10^ ^]^ The myotubes form during this stage become primary myofibers and work as a scaffold for secondary myofiber formation during the fetal stage.^[^ ^11^ ^]^ These, altogether, determine the post‐natal muscle development and its long‐term metabolic function.^[^ ^12^ ^]^

The expression of MRFs is regulated by essential regulatory factors, including other MRFs and non‐coding RNAs, with concomitant epigenetic modifications of their promoters.^[^ ^13^ ^]^ Distinct distal enhancers have been identified for *Myf5* and *Myod1*, which possess the binding motifs of regulatory factors.^[^ ^14^, ^15^, ^16^ ^]^ Consistently, *Myog* expression is initiated by MYOD1, a co‐transcription factor myocyte enhancer factor 2C (MEF2C), as well as regulated by myogenin itself as a positive feedforward loop, followed by the removal of inhibitory epigenetic marks in the promoter.^[^ ^13^, ^17^ ^]^ Available studies suggest that, MYOD1 can modulate 3D chromosome architecture to alter interactions between regulatory elements and target genes including *Myog*.^[^ ^18^, ^19^ ^]^ However, the dynamics of *Myog* expression and the involvement of possible cis‐regulatory elements and enhancer have not been defined.

The worldwide steady escalation of the obesity rate in reproductive‐age women over the past decades (currently ≈39.8% in the United States) has turned it into a pandemic.^[^ ^20^, ^21^, ^22^, ^23^, ^24^ ^]^ Maternal obesity (MO) not only affects maternal health but also exerts a negative impact on the development of their offspring, including myogenesis.^[^ ^25^, ^26^ ^]^ Recent studies, including our studies, have reported that MO induces a low‐grade chronic inflammation in the uterine microenvironment and upregulates nuclear factor kappa B (NF‐kB) signaling in embryos.^[^ ^25^, ^27^, ^28^ ^]^ As effectors of NF‐kB signaling, the NF‐kB family consists of RelA (P65), c‐Rel, RelB, P50 (NFKB1), and P52, which form RelA‐P50 and RelB‐P52 heterodimers to activate gene expression.^[^ ^29^ ^]^ As the key mediatory pathway of inflammation, NF‐kB signaling is involved in various pathophysiological processes, affecting myoblast proliferation and myogenic differentiation.^[^ ^30^, ^31^ ^]^ In satellite cells, NF‐kB enhances the cell cycle progression from G1 to S phase, while inhibiting myogenic differentiation.^[^ ^32^ ^]^ However, the impact of NF‐kB in embryonic myogenesis has yet to be explored, partially due to the technical challenges for studying early embryos. Using single‐cell (sc) multi‐omics, we explored the effects of inflammation and its effector, RelA, in impairing embryonic myogenesis due to MO.

We conducted scRNA and scATAC sequencing of E11.5 and E13.5 embryos, and spatial transcriptomic profiling of the myotome of E13.5 embryos from control and obese mice. We found that RelA binds with a novel distal enhancer that also possesses binding motifs of MYOD1, MEF2C, and MYOG, which suppresses *Myog* expression in embryos due to MO. Both in vitro knockdown and in vivo inhibition of RelA rescued embryonic myogenesis. These studies suggest that RelA and the associated NF‐kB signaling is a potential therapeutic target to rescue embryonic myogenesis in offspring from mothers with obesity and other inflammatory conditions.

## 2 Results

### 2.1 Single‐Cell Transcriptomic and ATAC Sequencing of the Mouse Embryos

**Figure 1:**
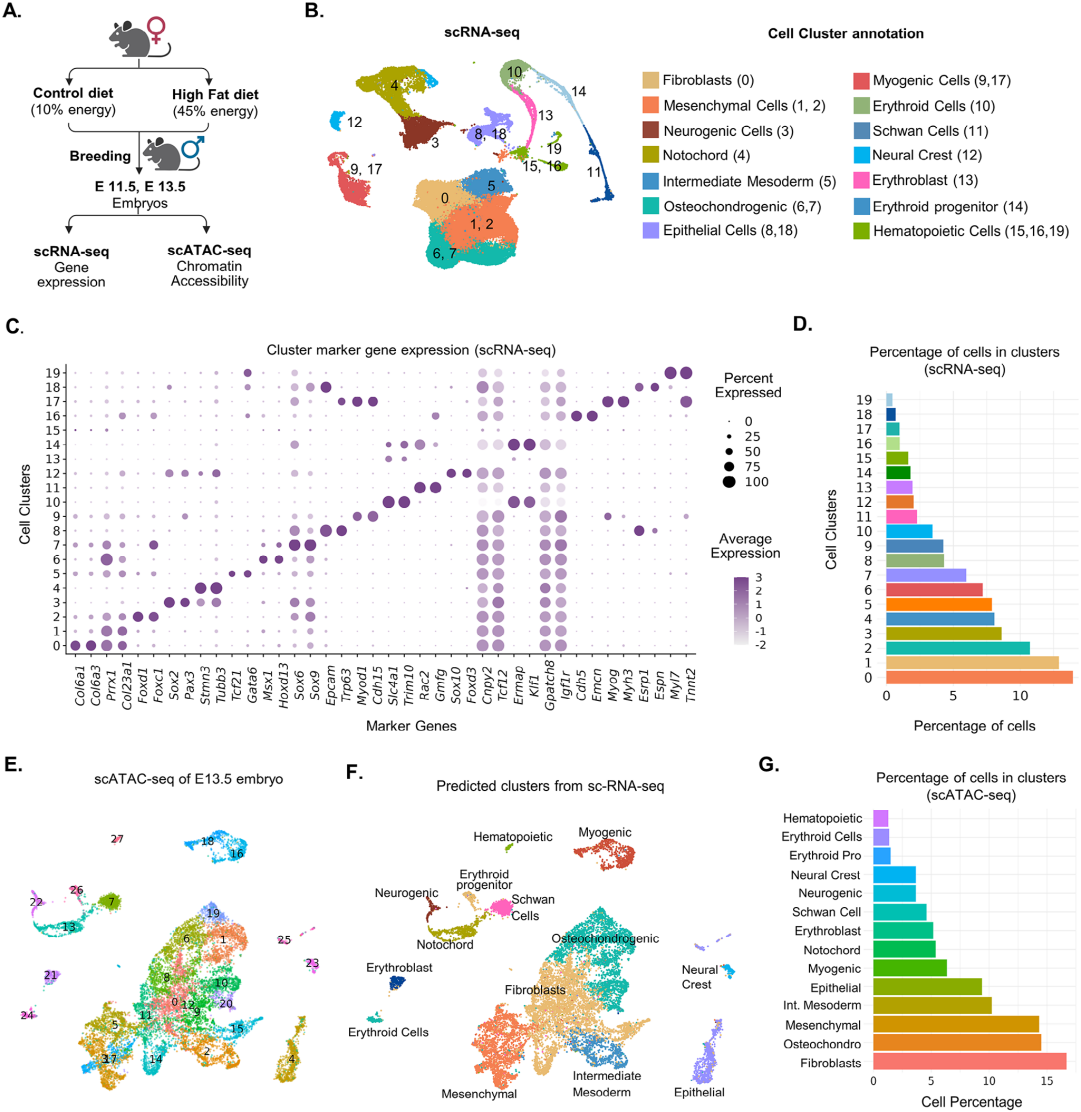
Single‐cell RNA and ATAC‐seq of embryos from control (CT) and obese (MO) mice. A) Schematic diagram of experimental design for induction of obesity in female mice and scRNA‐seq of E11.5 & E13.5 embryos and scATAC‐seq of E13.5 embryos. B) Uniform manifold approximation and projection (UMAP) plot showing 20 major clusters from the scRNA‐seq data sets of both time points. Names of individual clusters are listed next to the figure along with their corresponding identification number. C) Dot plot showing top differentially expressed lineage‐specific markers across all the 20 clusters identified from the integrated scRNA‐seq dataset. D) Percentage of each cell cluster in the integrated scRNA‐seq data. E) UMAP plot of scATAC‐seq showing 27 cell clusters from E13.5 embryos of CT and MO mice. F) Predicted cell clusters in scATAC‐seq from the scRNA‐seq by cross‐modality integration and label transfer. G) The percentage of each predicted cell cluster in the integrated scATAC‐seq data.

To induce obesity, we randomly divided female mice into control (CT) and MO groups. CT group was exposed to a control diet containing 10% energy from fat, and the MO group was exposed to a High‐fat diet (HFD) containing 45% energy from fat until there was a 20% difference in the body weight between the two groups in ≈10 weeks (**Figure** **1**A; Figure S1A, Supporting Information). We also conducted an intraperitoneal glucose tolerance test (IpGTT) and fasting blood glucose test, where obese mice exhibited higher fasting blood glucose and less glucose tolerance (Figure S1B, Supporting Information).

During the first wave of myogenesis, myo‐progenitor cells highly express *Myf5* to initiate myogenic commitment, and *Myog* expression escalates later and reaches its peak at E13.5 for terminal differentiation of myoblasts and their fusion to form myotubes.^[^ ^33^ ^]^ To investigate the impact of MO on myoblast differentiation and myotube formation, we conducted scRNA‐sequencing of embryonic tissue at E11.5 and 13.5 and scATAC‐seq at E13.5 from C57BL/6 control and obese female mice. For scRNA‐seq data processing, after filtering out the low‐quality cells, we recovered a total of 43,813 cells from where 27,359 cells were from E11.5 to 18,454 cells from the E13.5 group. Using Seurat V5, we combined all the cells from two developmental time points and clustered them following the non‐linear dimensional reduction technique: UMAP. We identified 20 different cell clusters from our integrated data set and annotated them based on the expression of specific marker genes (Figure 1B; Tables S1 and S2, Supporting Information). Among the major cell clusters, cluster 0 was embryonic fibroblast cells, expressing fibrogenic marker genes *Col6a1, Col6a3, Col1a1*, and *Pdgfra*, accounting≈15% of all cells; clusters 1 and 2, two next major clusters, accounted for ≈13% of cells and expressed embryonic mesenchymal marker genes *Prrx1* and *Twist1* (Figure 1C,D; Table S1, Supporting Information).^[^ ^34^, ^35^ ^]^ On the other hand, clusters 9 and 17 expressed myogenic markers *Myod1* and *Cdh15*; clusters 6 and 7 highly expressed osteochondrogenic marker genes *Msx1* and *Sox9* (Figure 1B,C; Table S1, Supporting Information).^[^ ^26^, ^36^ ^]^

With the progression of embryonic days from E11.5 to E13.5, there was a significant decline in the mesenchymal cell population (clusters 1 and 2), and a gradual increase in the fibrogenic (cluster 0), osteochondrogenic (clusters 6 and 7) and myogenic cells (clusters 9 and 17; Figure S1C,D, Supporting Information). The gradual decrease in mesenchymal progenitor cell population and concomitant increase in the lineage‐specific cell types showed an ongoing progression of organogenesis and illustrated a landscape of embryonic development during E11.5 and E13.5.^[^ ^37^ ^]^

In scATAC‐seq, we recovered ≈16 283 cells after initial pre‐processing and quality control to remove the low‐quality cells. Clustering and UMAP visualization of those cells by following non‐linear dimension reduction methods using Signac 2.1.0, we identified 27 unique cell clusters (Figure 1E). To interpret the cell type, we conducted the cross‐modality integration and label transfer from our scRNA‐seq data and predicted the cell types of scATAC‐seq clusters (Figure 1F). Major cell clusters such as neurogenic, chondrogenic, and myogenic clusters had very high (>90%) prediction scores, while the mesenchymal and fibrogenic cell clusters had a comparatively low prediction score (<80%) which could be due to their heterogeneity during this developmental stage (Figure S1E, Supporting Information). The predicted clusters exhibited similar gene activity patterns when compared to the marker gene expression from scRNA‐seq (Figure S1F, Supporting Information). For instance, the fibrogenic cluster (Cluster 0) had higher expression of the *Col6* gene in both scRNA and scATAC‐seq data; the myogenic clusters 17 and 9 expressed *Myod1, Cdh15, Myog*, and *Myh3* for both scRNA and scATAC‐seq (Figure S1F, Supporting Information). Similar to scRNA‐seq, mesenchymal cell cluster (cluster 1) and fibrogenic cell cluster (cluster 0) possessed the majority of cells in scATAC‐seq (Figure 1G). We identified 266 959 variable peaks across the cells, of which 41% overlapped with the promoter, 15% with exons, and 28% with introns and intergenic regions (Figure S1G, Supporting Information). Altogether, our scRNA and scATAC‐seq captured the landscape of early embryonic development and the developmental trajectories of myogenic, osteogenic, and fibrogenic cells.

### 2.2 Myogenic Developmental Trajectory

**Figure 2:**
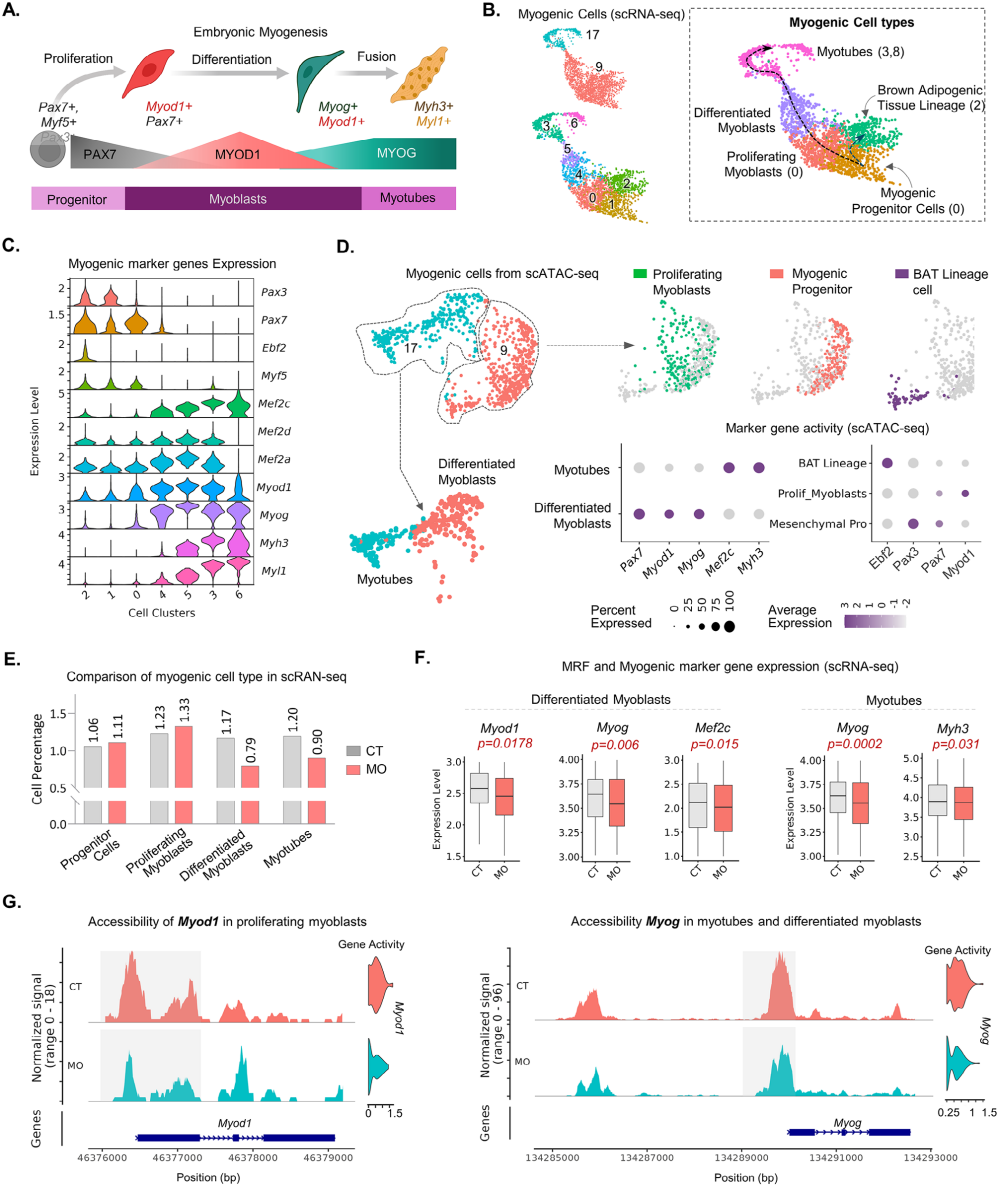
MO inhibits embryonic myogenesis via inhibition of *Myog* expression. A) Schematic diagram illustrating the gradual progress of embryonic myogenesis from myogenic progenitor cells to myotube formation under the regulation of different myogenic regulatory factors. B) Isolation of myogenic cluster and reconstruction of myogenic development trajectory from integrated scRNA‐seq of E11.5 and E13.5 embryos. C) Myogenic cell type‐specific marker gene expression in the reconstructed developmental trajectory. D) Identification of myogenic cell types from E13.5 scATAC‐seq based on the marker gene activity. E) Comparison between percentages of myogenic cell populations from scRNA‐seq. F) Comparison of myogenic marker genes and myogenic regulatory factors in differentiated myoblasts and myotubes from integrated scRNA‐seq. G) Accessibility and activity of myogenic marker genes in myogenic cell population from scATAC‐seq.

During early embryonic development, the myotome‐derived *Pax3, Pax7*, and *Myf5+* myogenic progenitor cells differentiate into myocytes and fuse to form *Myh3, Myl1+* multinucleated myotubes under the governance of MRFs *Myf5, Myod1 and Myog* (**Figure** **2**A). Cell clusters 9 and 17 from our scRNA‐seq expressed those marker genes and MRFs (Figure S2A, Supporting Information). Therefore, we extracted all cells from both clusters and subclustered them into 7 unique clusters (clusters 0 to 6) to reconstruct the myogenic developmental trajectory (Figure 2B). During embryonic myogenesis, the PAX3+ mesenchymal progenitor cells are the first cells to appear from the dorsomedial lips of dermomyotome that express *Pax7* to form myotome, which was identified in cluster 1 (Figure 2C).^[^ ^8^ ^]^ MYF5 is the earliest MRF that determines their myogenic lineage specification.^[^ ^7^ ^]^ MYOD1 is the later transcription factor that starts expressing ≈E10.5 and overlapping *Myf5* expression which were identified in cluster 0 (Figure 2B,C). *Myod1* expression induces differentiation of myoblasts into committed myogenic lineage cells and peaked among cells of clusters 4 and 5 (Figure 2B,C).^[^ ^38^ ^]^ MYOG is the last regulatory factor in embryonic myogenesis required for the fusion of mononucleated myoblasts to multinucleated *Myh3* and *Myl1+* myotubes which were observed in clusters 3 and 6 (Figure 2B,C).^[^ ^39^ ^]^ Brown adipocytes, contributed by multiple tissue lineages including *Pax3* and *Myf5+* mesenchymal progenitor cells, are positive for *Ebf2* which were present in cluster 2 (Figure 2B,C).^[^ ^8^ ^]^

Additionally, compared to E11.5, E13.5, myogenic cells exhibited a lower prevalence of progenitor cells and a higher prevalence of differentiated myoblasts and myotubes (Figure S2B, Supporting Information). The expression of *Pax3*, a marker of progenitor cells, was not identifiable at E13.5, while *Myh3* and *Myl1* were highly expressed in E13.5 myogenic cells (Figure S2B, Supporting Information).

To identify similar cellular heterogenicity in the myogenic cell population from our scATAC‐seq at E13.5, we extracted cells from clusters 9 and 17. Both clusters exhibited higher activity and accessibility of myogenic marker genes P*ax7, Myod1, Myog*, and *Myh3* (Figure 2D; Figure S2C, Supporting Information). Further clustering of both clusters 9 and 17 exhibited 5 sub‐clusters. Two sub‐clusters from cluster 17 exhibited higher activities of *Myod1*, *Myog*, *Myh3*, and *Myl1*, which denotes as differentiated myoblasts and multinucleated myotubes respectively (Figure 2D; Figure S2D, Supporting Information). On the other hand, three sub‐clusters from predicted cluster 9 expressed *Pax3*, *Pax7, Myod1*, and *Ebf2* that identified them as the mesenchymal progenitors, proliferating myoblasts, and BAT lineage cells, respectively (Figure 2D; Figure S2E, Supporting Information). Additionally, *Myod1* was only found in proliferating myoblasts not in the other two clusters (Figure 2D).

Cumulatively, these data identify the heterogenic cell types present in the myogenic cell populations of embryos and portray the gradual progression of myogenic developmental trajectory based on their gene expression and chromatin accessibility. In addition, a portion of BAT progenitor cells were also identified with a mesenchymal progenitor origin.

### 2.3 *Myog* and Myotube Formation were Inhibited in Differentiated Myoblasts due to Maternal Obesity

The commitment of progenitor cells to the myogenic lineage and their fusion to multinucleated myotubes are two major steps of myogenesis. We compared the expression and accessibility of MRF genes between cells from CT and MO groups in both scRNA and scATAC‐seq (Tables S3 and S4, Supporting Information) Combined E11.5 and E13.5 scRNA‐seq data didn't exhibit difference in the expression of *Myf5* and other early myogenic marker genes such as *Pax7* (Figure S2F, Supporting Information). Similarly, the expression of *Ebf2*, the earliest gene expressed in the BAT progenitor cells didn't differ (Figure S2G, Supporting Information). We observed a lower expression of *Myod1* in proliferating myoblasts, however, the difference was not significant and percentage of cells didn't differ between the two groups (Figure 2E; Figure S3A, Supporting Information). MO impaired the expression of *Myod1* and *Myog* in the differentiated myoblast cells (Figure 2F; Figure S3B, Supporting Information). The markers of the terminal stage of embryonic myogenesis, *Myog, Myh3* and *Myl1* were expressed much lower in myotubes from MO group (Figure 2F; Figure S3B, Supporting Information). The percentages of differentiated myoblasts and myotubes were also ≈32% and ≈25% lower in MO embryos, respectively (Figure 2E). In term of embryonic days, *Myod1* and *Myog* both were downregulated in myoblasts and myotubes during E11.5 however only *Myog* exhibited lower expression in both cell types during E13.5 (Figure S3C, Supporting Information). Additionally, gene ontology (GO) analysis of the downregulated genes identified their association with major biological processes including “Muscle cell differentiation”, “Myoblast Differentiation”, “Myoblast fusion” and “Myotube cell Development”, suggesting the downregulation of myogenic differentiation and myotube formation. (Figure S3D, Supporting Information). In short, the negative impact of MO on embryonic myogenesis was highly evident in the differentiated myoblasts and myotubes.

In our scATAC‐seq at E13.5, the number of cells in both myogenic clusters from the CT and MO groups was comparable (Figure S3E,F, Supporting Information). Gene accessibility profiles from our scATAC‐ seq also exhibited a similar impact of MO on the myogenic developmental trajectory. The accessibility of *Pax7* and *Myf5* in the myogenic progenitor cell population and *Ebf2* from brown adipocyte lineage didn't vary between the progenitor cell populations from CT and MO groups along with their gene activity (Figure S3G,H, Supporting Information). However, the accessibility of *Myod1* differed in the proliferating myoblasts between the two groups (Figure 2G). Furthermore, *Myog* accessibility in the differentiated myoblasts and myotubes especially in the promoter region was lower in MO (Figure 2G).

Taken together, the scRNA and scATAC‐seq analysis of embryonic tissue samples showed that MO impairs *Myog* expression and embryonic myogenesis, mainly during the terminal differentiation and myotube formation.

### 2.4 Maternal Obesity Induces an Inflammatory Response and Activates NF‐kB Signaling in Embryos

**Figure 3:**
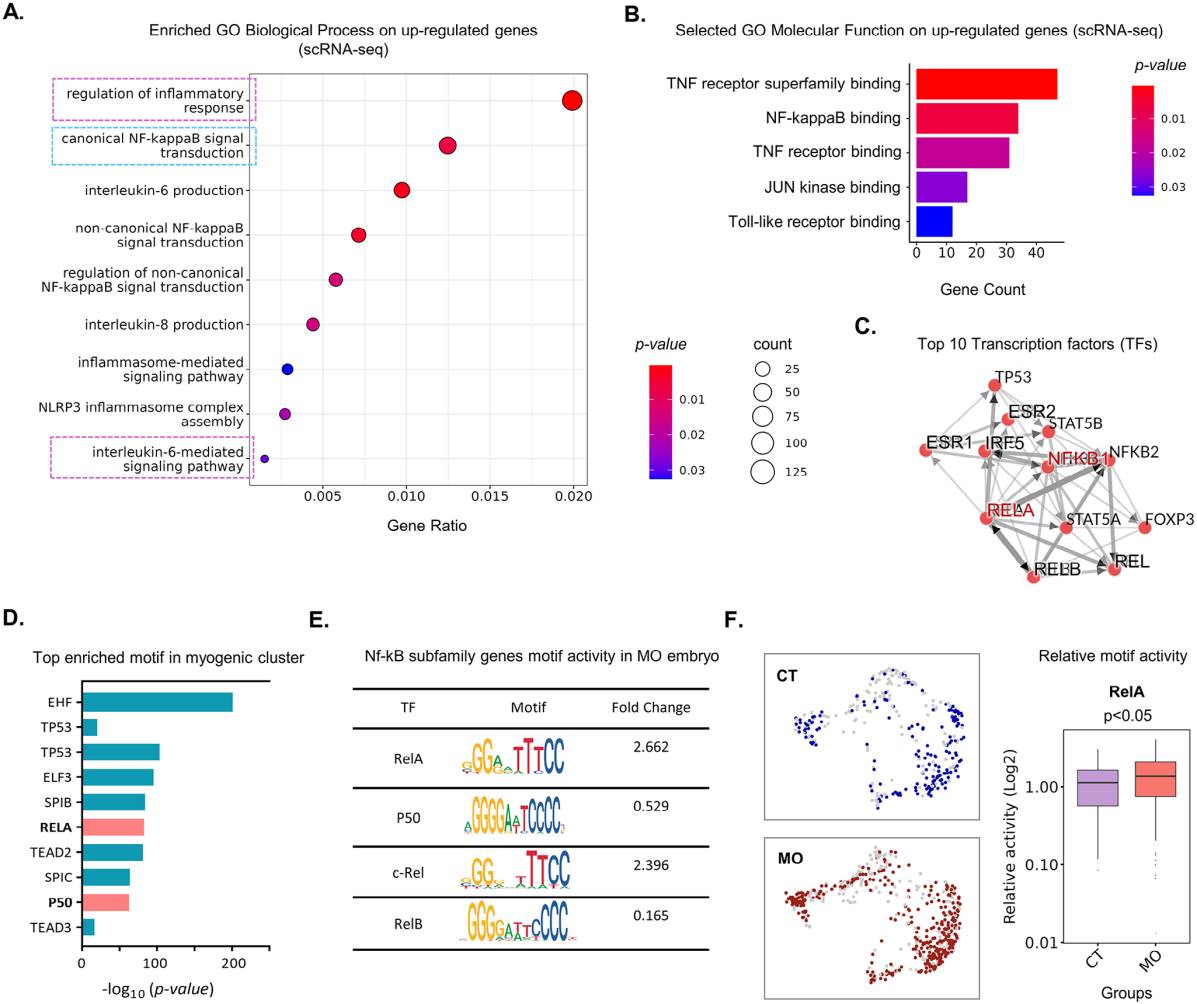
MO induces an inflammatory response and activates NF‐kB signaling in embryos. A) Enriched GO biological processes associated with the upregulated genes in MO embryos. B) Enriched GO molecular function associated with the upregulated genes in MO embryos. C) Top ten transcription factors regulating upregulated genes in myogenic clusters from scRNA‐seq. D) Top ten enriched motifs in myogenic cell clusters from scATAC‐seq. E) Motif activity and fold changes of NF‐kB family genes in MO embryos. F) Comparative motif activity of RelA in myogenic clusters from CT and MO embryos.

Maternal obesity elevates pro‐inflammatory cytokine levels in maternal circulation and induces an inflammatory response in the embryos.^[^ ^27^, ^40^ ^]^ To explore the impact of MO on inflammatory response in embryos, we conducted the Gene Ontology (GO) analysis of upregulated genes from the DEGs between CT and MO embryos. The GO analysis suggests the association of upregulated genes in MO with biological processes “Regulation of inflammatory response”, “Canonical NF‐kB signaling”, “IL‐6 and IL‐8 production”, and “IL‐6 mediated signaling pathway” (**Figure** **3**A; Figure S4A,B, Supporting Information). In addition, GO analysis identified the involvement of upregulated genes in MO with molecular functions: “TNF receptor superfamily binding”, “NF‐kB binding”, and “TNF receptor binding” (Figure 3B; Figure S4C, Supporting Information). Additionally, analysis of transcription factors (TF) associated with the upregulated genes by ChEA3 identified members of the NF‐kB family (NFKB1, RelA) among the top associated TF lists. (Figure 3C). These data showed the up‐regulation of inflammation in MO embryos.

NF‐kB family, consisting of RelA, c‐Rel, RelB, P50, and P52, exists as RelA homodimer, RelA‐P50, and RelB‐P52 heterodimers when activated,^[^ ^29^ ^]^ mediating the canonical and non‐canonical NF‐kB signaling pathways respectively.^[^ ^30^ ^]^ Proinflammatory cytokines, including TNF‐a (tumor necrosis factor‐alpha), IL‐1 (interleukin‐1), IL‐6, and IFN‐I (type I interferons) are major activators of canonical NF‐kB signaling.^[^ ^31^ ^]^ However, the expression of the NF‐kB family genes didn't alter due to MO (Figure S4D, Supporting Information).^[^ ^32^, ^41^ ^]^ In canonical NF‐kB signaling, RelA homodimer or RelA and P50 heterodimer form a complex with IκBα (inhibitor of nuclear factor kappa B) and remain inactive. Upon activation by different external stimuli, IκB kinase (IKK) phosphorylates IκBα and triggers its degradation by ubiquitination. This process leads to the rapid nuclear translocation of the canonical RelA dimer and triggers the expression of their targeted genes.^[^ ^42^ ^]^ To further identify the most active transcription factors of the NF‐kB family, we performed motif enrichment analysis in myogenic cell clusters based on scATAC‐seq (Table S5, Supporting Information). RelA (P65) and P50 (NFKB1) were among the top active motifs in the myogenic cells (Figure 3D). Additionally, among the members of the NF‐kB family, RelA motif activity was upregulated (Fold change: 2.26) in the MO embryos (Figure 3E), which was confirmed by the differential motif activity analysis (Figure 3F; Figure S4E–G, Supporting Information). In short, MO activates NF‐kB signaling in myogenic cells demonstrated through the upregulation of RelA dimer activity.

### 2.5 Spatial Transcriptomic Profiling of Myotome Reveals Myog Inhibition and NF‐kB Upregulation Due to MO

**Figure 4:**
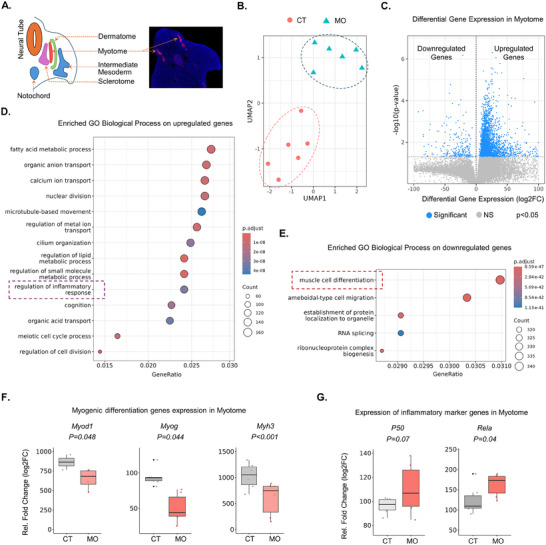
Spatial transcriptomic sequencing of embryonic myotome from CT and MO embryos. A) Fluorescence image of embryonic myotome in cross‐section of embryos visualized by MYH3+ cells. B) UMAP plot of principal component analysis (PCA) of embryonic myotome from CT and MO embryos. C) Volcano plot of differentially expressed genes in the myotome of MO group compared with CT group. D) Enriched GO biological processes associated with the upregulated genes in embryonic myotome. E) Enriched GO biological processes associated with the downregulated genes in embryonic myotome. F) Relative mRNA abundance of myogenic regulatory factors and marker genes in myotome between CT and MO embryos. G) Relative mRNA abundance of members from NF‐kB family in myotome between CT and MO embryos.

To gain deep insight into the embryonic myotome due to MO, we conducted GeoMx DSP transcriptomic profiling of myotome (**Figure** **4**A; Figure S5A, Supporting Information) and principal component analysis (PCA) grouped samples from CT and MO into distinct clusters, indicating differences in their transcriptional profiles. (Figure 4B). A list of down‐regulated genes and up‐regulated genes were identified between CT and MO embryos (Figure 4C; Figure S5B, Supporting Information). Similar to scRNA‐seq, the GO analysis with the differentially expressed genes revealed the association of the biological process “Regulation of inflammatory response” with the up‐regulated genes and “Muscle cell differentiation” with the down‐regulated genes (Figure 4D,E). Furthermore, significant down‐regulation of *Myod1* and *Myog*, as well as *Myh3* was observed in the MO embryos (Figure 4F). On the other hand, *Pax7* was higher in MO embryos, whereas *Pax3* and the levels of *Mef2a*, and *Mef2c* didn't differ (Figure S5C, Supporting Information). In terms of canonical NF‐kB signaling, a higher relative abundance of *RelA* and *P50* was observed in the MO group (Figure 4G; Figure S5D, Supporting Information).

Overall, similar to our findings from scRNA and scATAC‐seq, the GeoMx spatial transcriptional profiling confirmed the suppression of myogenesis and the up‐regulation of NF‐kB family members *RelA* and *P50* due to MO in the myotome.

### 2.6 NF‐kB Binds to an Enhancer of Myog to Down‐Regulate Its Expression

**Figure 5:**
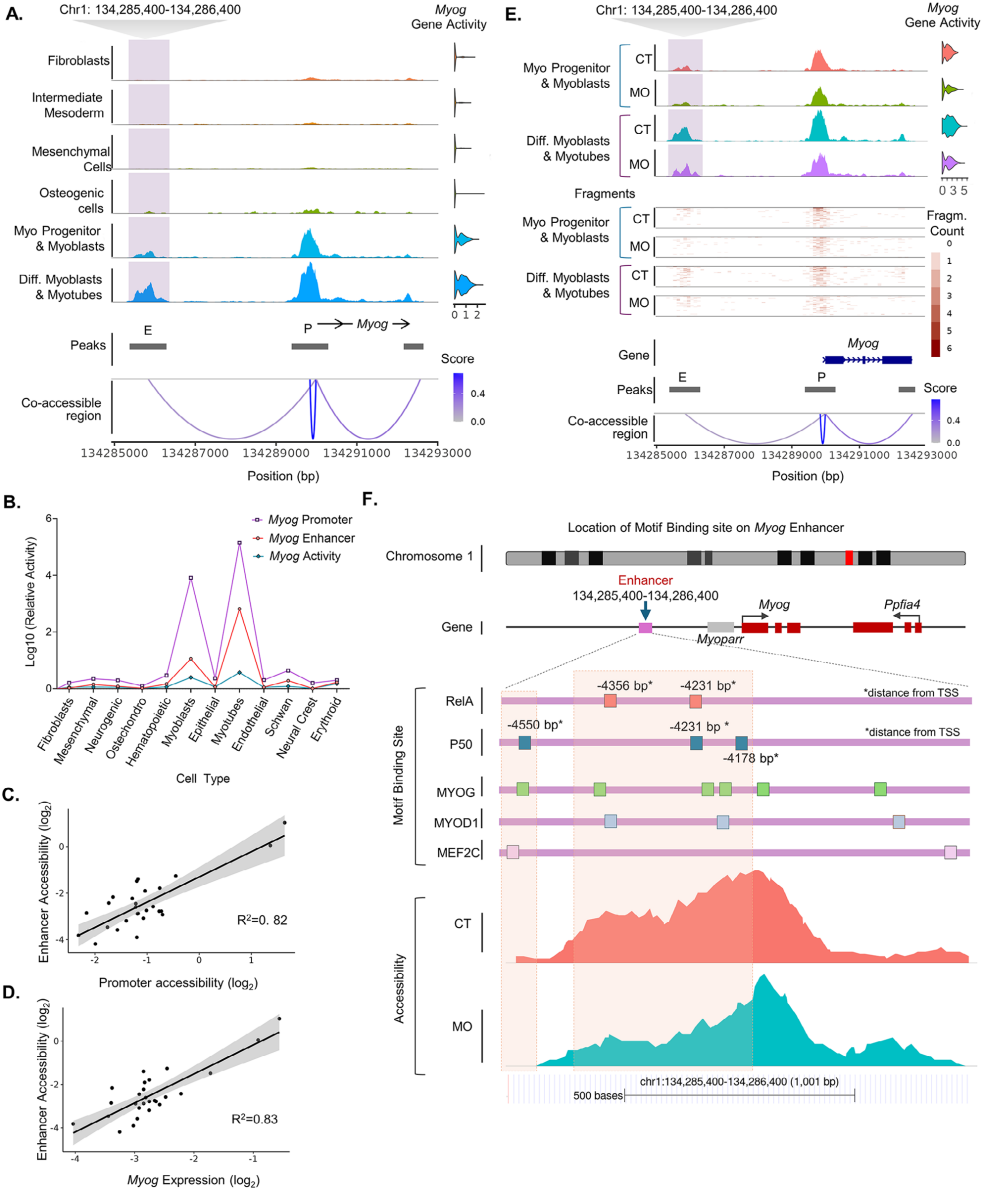
A regulatory enhancer region regulates *Myog* promoter activity and is inhibited by RelA binding. A) A distal co‐accessible enhancer region (E) associated with the cell type specific activity of the *Myog* promoter (P). B) Comparison of relative activity of co‐accessible enhancer region along with *Myog* promoter and gene activity. C) Correlation between the *Myog* enhancer and promoter accessibility. D) Enhancer accessibility and *Myog* expression from scATAC‐seq of E13.5 embryos. E) Relative comparison of *Myog* enhancer accessibility and subsequent impact on its promoter and gene activity in scATAC‐seq from CT and MO embryos at E13.5. F) Location of RelA, P50, MYOG, MYOD1 and MEF2C binding motifs in the *Myog* enhancer region and relative accessibility from scATAC‐seq of CT and MO embryos.

Enhancers are *cis*‐regulatory regions that enhance the expression of nearby genes.^[^ ^43^ ^]^ The promoter‐enhancer interaction, i.e., cis‐regulatory interactions can be identified from scATAC‐seq data by analyzing the *cis*‐co‐accessible networks (CCANs).^[^ ^44^ ^]^ CCAN analysis of our current scATAC‐seq datasets revealed a co‐accessible region (Chr1: 134285400‐134286400) proximal to the *Myog* promoter (**Figure** **5**A). The accessibility of this co‐accessible enhancer region was cell‐type specific and was only found in myogenic cell clusters (Figure 5A). Also, the activity of this enhancer region followed a similar pattern of changes as the *Myog* promoter and its expression (Figure 5B). The accessibility of this enhancer region was highly correlated with *Myog* promoter accessibility (R^2^ = 0.82) and gene activity (R^2^ = 0.83) (Figure 5C,D). In alignment with this finding, the accessibility of this identified enhancer region was lower in both MO myoblasts and myotubes where *Myog* expression was inhibited (Figure 5E). This enhancer region possesses several binding sites of NF‐kB family members: RelA, P50, and MYOD1and MYOG, and their co‐factor MEF2C from JASPER database (Figure 5F; Figure S6A, Supporting Information).^[^ ^45^ ^]^ In ReMap, a database of regulatory regions based on DNA binding experiments like ChIP‐seq, exhibits active MYOG and MYOD1 binding activities in this region in C2C12 cells, embryonic myoblasts and myotubes (Figure S6B, Supporting Information). We observed lower accessibility of the MYOD1, MYOG, and MEF2C binding sites on the enhancer region, as well as their lower motif binding activity in MO myogenic cells (Figure 5F; Figure S6C–E, Supporting Information). We didn't observe changes in the motif binding activity of other MRFs (Figure S6E,F, Supporting Information).

Together, we identified an enhancer region of *Myog* ≈4 kb upstream of its promoter, which is highly correlated with its promoter activity and expression. Additionally, the presence of a binding site on the enhancer and upregulation of motif activity suggest enhanced recruitment of RelA dimer to the enhancer region and the down‐regulation of *Myog* expression by inhibiting the enhancer function in MO embryos.

### 2.7 Binding of RelA to the Myog Enhancer Inhibits Its Expression and Impacts Myotube Formation

**Figure 6:**
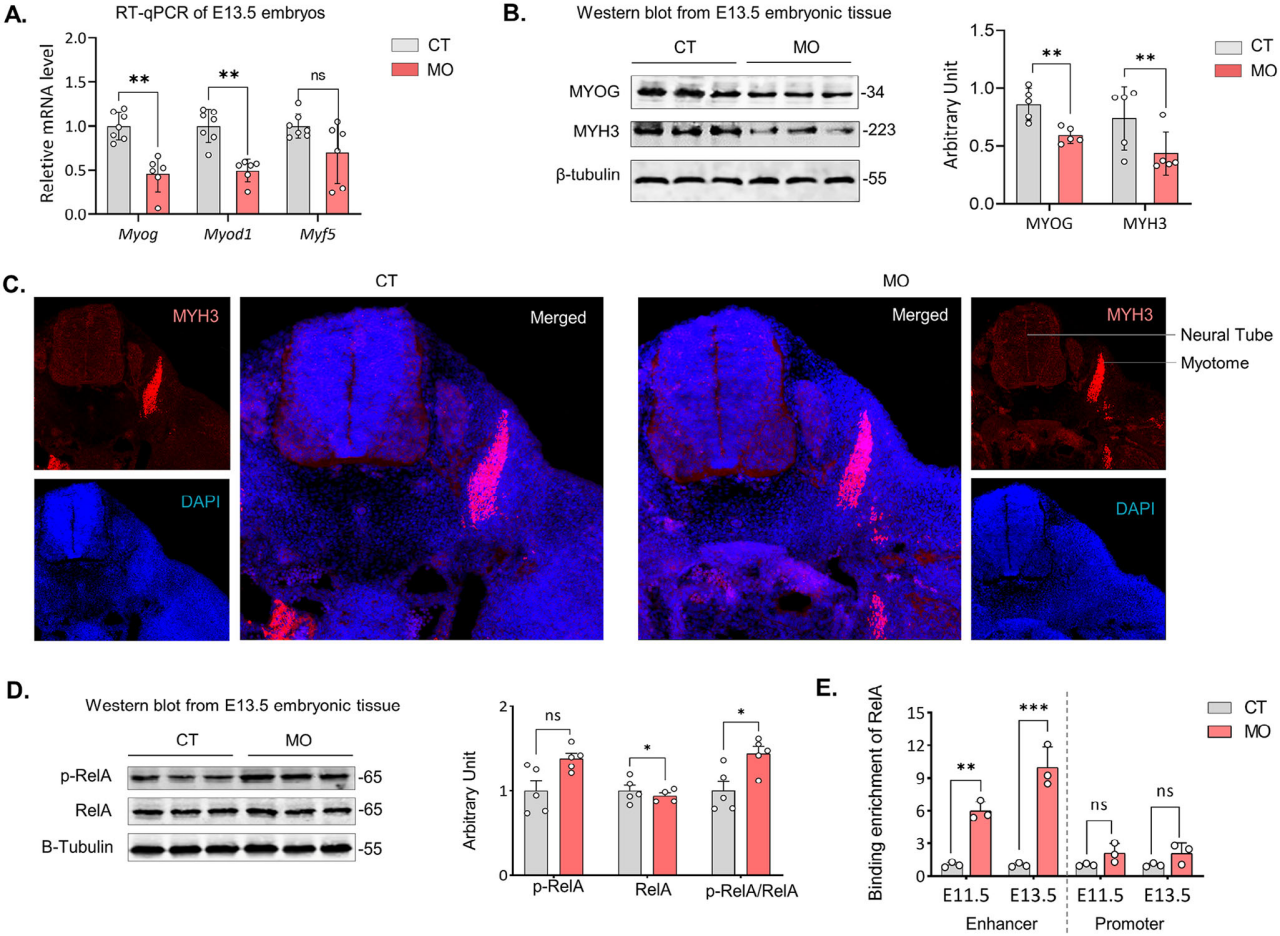
Inhibition of *Myog* impaired myogenesis E13.5 embryonic tissues. A) Relative mRNA expression of myogenic regulatory factors *Myog, Myod1* and *Myf5* in embryos from CT and MO. B) Cropped Western Blots of MYOG and MYH3 (β‐Tubulin as loading control). C) Fluorescence image of embryonic myotome at E13.5 from CT and MO embryos visualized by MYH3+ cells. D) Cropped Western Blots of p‐RelA, and RelA (β‐Tubulin as loading control). E) Binding enrichment of RelA with the enhancer and promoter region of *Myog* from CT and MO embryos during E11.5 and E13.5. CT: Control group; MO: Maternal Obesity group. Data are shown as mean ± SEM, and each dot represents one litter (*n* = 5). ^∗^ *p* < 0.05, ^∗∗^ *p* < 0.01, and ^∗∗∗^ *p* < 0.001 in CON versus MO by one‐tailed unpaired Student's *t*‐test (a–e).

To confirm our findings from scRNA, scATAC‐seq, and spatial sequencing, we conducted RT‐qPCR analysis of E13.5 CT and MO embryonic tissue samples. The expression of *Myod1* and *Myog* was lower in MO embryos (**Figure** **6**A). Similarly, the protein levels of MYOG and MYH3 were lower in MO embryos, which aligns with the findings from scRNA and spatial Seq (Figure 6B). However, the mRNA expression of *Myf5* did not deviate in the MO embryo (Figure 6A). In addition, immunofluorescence staining of E13.5 embryos with MYH3 antibody showed a smaller myotome in the cryosections of MO embryos relative to CT embryos (Figure 6C).

RelA is a key mediator of canonical NF‐kB signaling, and its phosphorylation is an indication of its active state, which enhances its nuclear translocation.^[^ ^31^ ^]^ E13.5 embryos from MO mice had a higher ratio of p‐RelA/RelA (Figure 6D). By ChIP assay using an antibody against RelA, we found higher recruitment of RelA to the enhancer region of both E11.5 and E13.5 MO embryos (Figure 6E), which might suppress the binding of other transcription factors and inhibit *Myog* expression.

Together, these analyses verify our findings from scRNA, scATAC and spatial seq that MO elevated the binding of RelA dimer to the enhancer region of *Myog* and inhibition of myogenesis.

### 2.8 *Myog* Expression and Myotube Formation are Impaired in Differentiated Myoblasts Due to RelA

**Figure 7:**
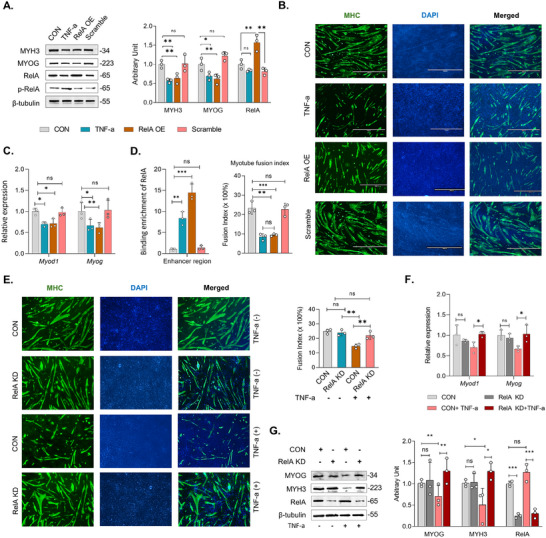
RelA inhibits *Myog* and myotube formation in differentiated myoblasts from C2C12 cells in vivo. A) Cropped western blots of MYOG, MYH3, RelA and p‐RelA (β‐tubulin was used as loading control) B) Immunostaining of myosin heavy chain (MHC). C) Relative mRNA expression of myogenic marker gene *Myod1* and *Myog*. D) Binding enrichment of RelA at the distal enhancer region of *Myog* in C2C12 cells after activation of NF‐kB signaling activation by TNF‐a and RelA overexpression (RelA OE) by plasmid DNA. E) Immunostaining of myosin heavy chain (MHC). F) Relative mRNA expression of myogenic marker genes *Myod1* and *Myog*. G) Cropped western blots of MYOG, MYH3, and RelA (β‐tubulin was used as loading control) in C2C12 cells with/without siRNA‐mediated RelA knockdown and TNF‐a in C2C12 myoblast cells. CON: Control, RelA KD: RelA knockdown. Data are presented as mean ± SEM, and each dot represents one independent experiment (*n* = 3). ^∗^ *p* < 0.05, ^∗∗^ *p* < 0.01, and ^∗∗∗^ *p* < 0.001 groups by one‐way ANOVA (*n* = 3).

The nuclear translocation of the RelA homodimer, or a heterodimer of RelA with another member of the NF‐κB family, is required for the activation of NF‐κB signaling and for binding to the regulatory regions of target genes to exert its transcriptional effects.^[^ ^46^ ^]^ The release of sequestrated RelA dimer by phosphorylation and ubiquitination of IκBα triggers its rapid translocation to nucleus, however the overexpression of RelA is also associated with its nuclear positivity in both in vivo and in vitro condition, independent of its phosphorylation status in the canonical NF‐kB signaling cascade.^[^ ^47^, ^48^ ^]^ Therefore, to explore the role of RelA on myogenesis, we overexpressed RelA in C2C12 cell, as well as activated the NF‐kB signaling TNF‐a and evaluated their impact on myogenesis (Figure S7A, Supporting Information). We used scrambled plasmid with GFP as a positive control to evaluate the transfection efficiency and non‐specific effect on myogenesis (Figure S7B, Supporting Information). In the RelA overexpression (RelA OE) group, we observed a higher level of total RelA without changing its phosphorylation level, and the RelA level was unchanged in the TNF‐a group (**Figure** **7**A). Consistently, we observed lower levels of MYOG and MYH3 in t‐a and RelA OE groups (Figure 7A). Immunofluorescence imaging also exhibited a lower number of myotubes in both the RelA OE group and TNF‐a treated group (Figure 7B). Similarly, RT‐qPCR also showed the inhibition of *Myod1* and *Myog* expression in RelA OE and TNF‐a groups (Figure 7C). Activation of NF‐kB signaling by TNF‐a or RelA OE increased the translocation of RelA homodimer or RelA‐P50 heterodimer into the nucleus which increased their binding with enhancer region of *Myog* and inhibited myogenesis in C2C12 cells. Together, these data show that activation of canonical NF‐kB signaling impairs *Myog* expression and myotube formation.

To further verify the role of canonical NF‐kB signaling on myogenesis, we knocked down RelA by siRNA following the activation of NF‐kB signaling by TNF‐a in C2C12 cells (Figure S7C,D, Supporting Information). In the absence of TNF‐a, RelA KD only had minor effects on *Myod1*, and *Myog* expression, and myotube formation (Figure 7E,F). Consistently, NF‐kB activation by TNF‐a reduced the myotube formation in CON cells, which was absent in RelA KD cells (Figure 7E). Similarly, the mRNA levels of *Myod1* and *Myog* were not impacted in RelA KD cells after TNF‐a addition (Figure 7F). Knockdown of RelA didn't activate the canonical NK‐kB signaling in RelA KD cells and didn't exhibit deleterious effects on myoblast differentiation nor myotube formation (Figure 7G; Figure S7E, Supporting Information). Collectively, these data verify the crucial role of RelA in inhibiting *Myog* expression and myotube formation in C2C12 myogenic cells due to inflammation.

### 2.9 Metformin Treatment Attenuates NF‐kB Signaling and Rescues Myogenesis in MO Embryos

**Figure 8:**
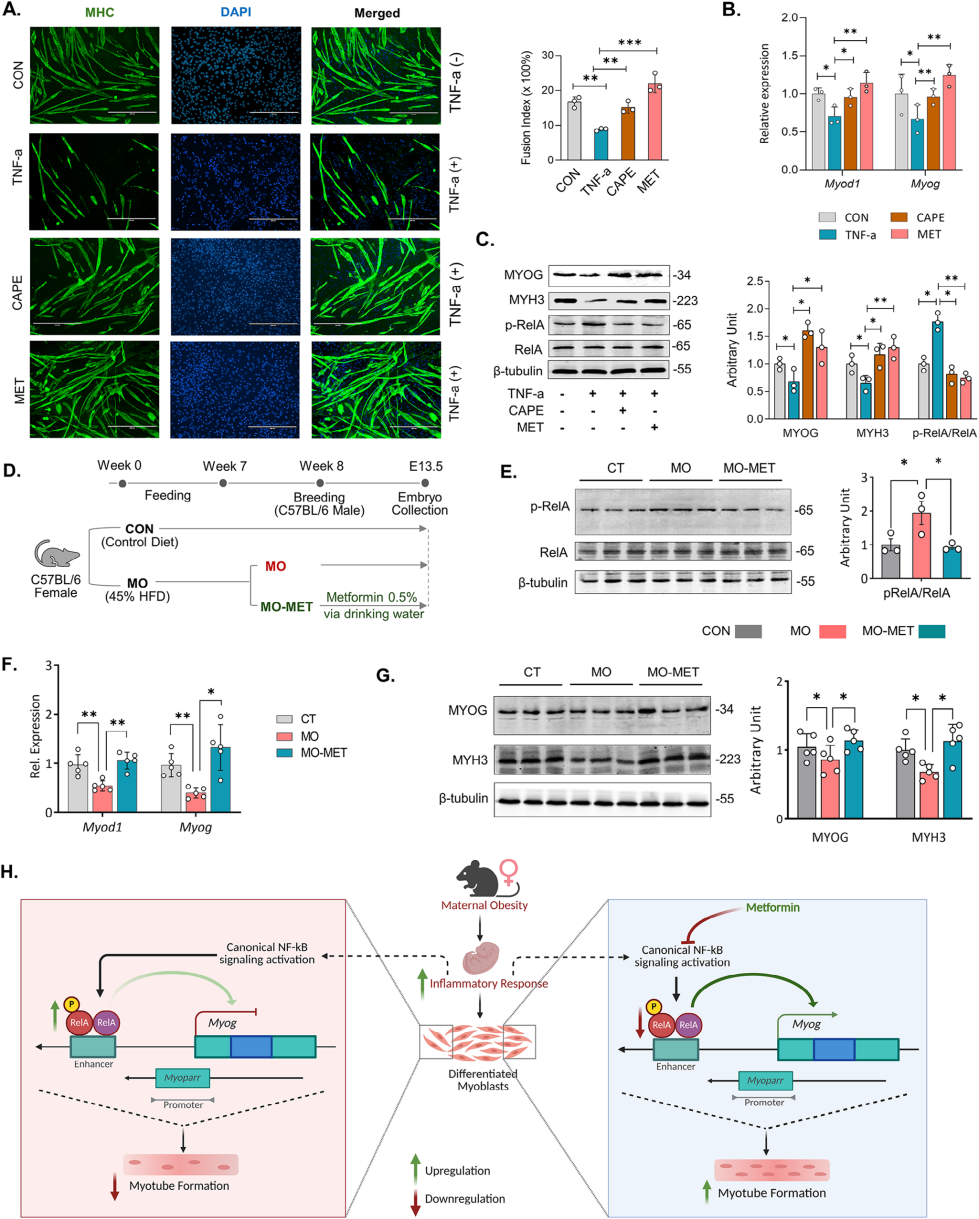
Metformin attenuates RelA activation and rescues *Myog* expression and myogenesis in vitro and E13.5 embryos. A) Immunostaining of myosin heavy chain (MHC). B) Relative mRNA expression of myogenic marker genes *Myod1* and *Myog*. C) Cropped western blots of MYOG, MYH3, p‐RelA and RelA (β‐tubulin was used as loading control) after activation of NF‐kB signaling by TNF‐a and its inhibition with CAPE (Caffeic Acid Phenethyl Ester) and MET (Metformin) treatments in C2C12 myoblast cells. D) Metformin treatment of obese mice and collection of E13.5 embryos. E) Cropped Western Blots of p‐RelA, and RelA (β‐Tubulin as loading control). F) Relative mRNA expression of myogenic regulatory factors *Myog* and *Myod1* in embryos. G) Cropped Western Blots of MYOG and MYH3 (β‐Tubulin as loading control) from CT (*n* = 6 mice per group), MO (*n* = 6 mice per group), and MO‐MET (*n* = 6 mice per group) groups. H) A schematic diagram summarizes the RelA dimer‐dependent mechanism impairing *Myog* expression and embryonic myogenesis due to maternal obesity. Data are presented as mean ± SEM, and each dot represents one litter. ^∗^ *p* < 0.05, ^∗∗^ *p* < 0.01, and ^∗∗∗^ *p* < 0.001.

Proinflammatory cytokines, including TNF‐a, IL‐1, IL‐6, and IFN‐I are major activators of canonical NF‐kB signaling.^[^ ^31^ ^]^ To explore the impact of NF‐kB activation in committed myoblasts and their myogenic differentiation, we used TNF‐a to induce inflammation in confluent C2C12 myoblasts (Figure S8A,B, Supporting Information). Furthermore, we used Caffeic Acid Phenethyl Ester (CAPE) and Metformin (MET) to inhibit the canonical NF‐kB pathway (Figure S8A, Supporting Information). CAPE specifically inhibits the ubiquitination and phosphorylation‐mediated activation of the RelA dimer and prevents their translocation into the nucleus to regulate their target genes.^[^ ^49^ ^]^ On the other hand, MET effectively inhibits the TNF‐a, HIF‐1a, and IL‐6‐mediated activation of inflammatory signaling in both AMP‐activated protein kinase (AMPK) and AMPK‐independent pathways.^[^ ^50^ ^]^ Immunofluorescence imaging exhibited a lower number of myotubes in TNF‐a treated group, on the other hand, the number of myotubes were increased after addition of CPAE and MET (**Figure** **8**A). Similarly, RT‐qPCR showed the inhibition of *Myod1* and *Myog* expression in TNF‐a group, which was rescued by MET and CAPE (Figure 8B). Activation of NF‐kB signaling by TNF‐a increased the p‐RelA/RelA ratio, which reduced MYOG and MYH3 contents in the myogenic cells (Figure 8C), and CAPE and MET rescued the expression of MYOG and MYH3 by reducing the p‐RelA/RelA ratio (Figure 8C). Notably, the number of differentiated myoblasts was comparatively lower in the TNF‐a group, indicating the negative impact of inflammation on myogenic differentiation (Figure S8C, Supporting Information). Together, these data show that activation of canonical NF‐kB signaling impairs *Myog* expression and myotube formation.

Metformin (MET) has long been used to control pre‐gestational and gestational diabetes, and prevailing data suggest additional health benefits of in‐utero exposure to offspring.^[^ ^51^, ^52^ ^]^ Therefore, we treated MO mice (Figure S8D, Supporting Information) with MET from one week before breeding to E13.5 for embryonic tissue collection (Figure 8E). Embryos from Met‐treated embryos didn't differ in their body weight (Figure S8E,F, Supporting Information), however, it reduced the inflammatory response in MO embryos as evidenced by the reduction of the p‐RelA /RelA ratio (Figure 8E). Excitingly, MET recovered *Myod1* and *Myog* expression in MO embryos (Figure 8F). Similarly, the protein contents of MYOG and MYH3 were elevated in MO embryos treated with Met (Figure 8G). In short, MET treatment of obese maternal mice suppressed inflammation and the canonical NF‐kB signaling in embryos, which rescued embryonic myogenesis (Figure 8H).

## 3 Discussion

The obesity pandemic in pregnant women is well documented to exert negative impacts on the development of skeletal muscle, including reduced muscle strength, endurance capacity, and smaller muscle mass of offspring.^[^ ^26^, ^53^, ^54^, ^55^, ^56^, ^57^ ^]^ The prenatal stage is critically important for skeletal muscle development.^[^ ^58^ ^]^ The primary myofibers form during the embryonic stage and lay the foundation for later muscle fiber development. Here, using multiomics tools, including scRNA‐seq, scATAC‐seq, and spatial transcriptomic profiling, we comprehensively analyzed the myogenic lineage specification and differentiation in mouse embryos. Our data suggest the existence of a distal cis‐regulatory enhancer region of Myog, which mediates the inhibition of embryonic myogenesis induced by MO.

Embryonic myogenesis can be separated into myogenic commitment and differentiation. During E11.5, we identified the downregulation of *Myod1* in myoblasts, but didn't observe a change in proliferating myoblast cell populations in MO embryos. Proliferating myoblasts also express *Myf5*, which precedes *Myod1* expression and rapidly downregulates during differentiation; *Myf5* and *Myod1* share similar binding sites and functionalities to induce the transcription of genes related to myogenic differentiation.^[^ ^59^ ^]^ We identified a lower percentage of differentiated myoblasts in the MO embryos.^[^ ^19^, ^60^ ^]^ More importantly, we observed downregulation of *Myog* in differentiated myoblasts at both E11.5 and E13.5 stages due to MO.^[^ ^9^ ^]^ We also identified a lower percentage of differentiated myoblasts and myotubes in the MO embryos. Consequently, our data suggest that the MO mainly affects the terminal differentiation and myotube formation during embryonic myogenesis while having less effect on the myogenic commitment and proliferation.

Expression of *Myog* is regulated by various transcription factors including MYF5 and MYOD1.^[^ ^10^, ^61^, ^62^ ^]^ These transcription factors bind to different conserved DNA binding motifs on the *Myog* promoter, which regulate its context‐dependent expression.^[^ ^62^ ^]^ In scATAC‐seq, we identified an enhancer region that is −4 kb upstream to the *Myog* TSS, and its location overlaps with a region recently confirmed as an active motif binding site by Self‐transcribing active regulatory region sequencing (STARR‐seq) in murine embryonic stem cells.^[^ ^63^ ^]^ Global Run‐on sequencing (GRO‐seq), STARR‐seq, and several other methods can measure enhancer activity, but have limitations in identifying the enhancer‐promoter pair.^[^ ^64^ ^]^ Our CCAN analysis identified the *Myog* enhancer‐promoter pair, which is highly correlated with *Myog* activity. Additionally, the accessibility of this enhancer region was highly cell‐type‐specific and only accessible in the myogenic cells. These enhancer regions also possess binding sites of transcription factors MYOD1, MEF2C, and even MYOG to regulate *Myog* expression. One of the potential roles of this enhancer region is likely to fine‐tune the *Myog* expression depending on cell states. During the decline of *Myod1* expression after the myogenic commitment, co‐transcription factors like MEF2C bind to these regions to up‐regulate *Myog* expression. Additionally, MYOG has its autoregulatory positive feedforward mechanism in combination with MEF2C.^[^ ^9^, ^65^ ^]^ We observed downregulation of MEF2C expression in both differentiated myoblasts and Myotubes, as well as lower activity in their binding sites on the enhancer due to MO, which might reduce the overall *Myog* expression. These observations in combined with other reports confirm the existence of an enhancer region that is correlated with *Myog* expression.

Obesity, metabolic diseases, and several pathological conditions in pregnant mothers including pelvic inflammatory disease (PID) and inflammatory bowel disease (IBD) can induce an inflammatory condition in the intrauterine microenvironment and activate inflammatory response in embryos.^[^ ^25^ ^]^ In this study, we found that RelA activity was highly up‐regulated among the members of the NF‐kB family due to MO and inhibition of RelA with Metformin both in vivo and in vitro rescued myogenesis. These data are concordant with in vitro studies that revealed increased myogenic activity in *RelA^−/−^* C2C12 cells and primary mouse embryonic fibroblast cells.^[^ ^31^ ^]^ Importantly, we discovered a novel enhancer containing RelA and P50 binding sites in the vicinity of *Myog* promoter and ChIP‐PCR confirmed higher binding of RelA dimer to the enhancer region in MO embryos. These findings suggest a potential mechanism leading to *Myog* down‐regulation during embryogenesis.

Metformin has been widely used in the treatment of gestational diabetes in women and comes with multiple benefits to both mothers and their embryos because of its ability to cross‐placenta.^[^ ^66^, ^67^ ^]^ Pre‐pregnancy and pregnancy treatment with metformin showed variable effects on offspring at different stages of their life. Periconceptional metformin treatment improves embryonic myogenesis which was associated with the inhibition of canonical NF‐kB signaling, consistent with our previous discovery that metformin suppresses fibrogenesis in E13.5 MO embryos.^[^ ^27^, ^68^ ^]^ Recent studies showed that metformin administration induced fetal growth restriction in murine and primate models during late gestation,^[^ ^69^, ^70^ ^]^ however, metformin administration during the first trimester has not been associated with developmental abnormalities or adverse outcomes at both fetal and adult stages.^[^ ^71^, ^72^, ^73^ ^]^ These results highlight the periconceptional use of metformin as a potential therapeutic agent to mitigate maternal inflammatory responses and improve embryonic myogenesis for obese mothers.

In summary, we found that MO impairs embryonic myogenesis via inhibition of *Myog* and terminal myogenic differentiation. Canonical NF‐kB signaling is a key mediator that inhibits *Myog* expression through the recruitment of RelA dimer to the enhancer region. Our findings also show that NF‐kB is a potential therapeutic target to rescue embryonic myogenesis and prevent the long‐term impact of MO on offspring health.

## 4 Experimental Section

### Mice

Wild‐type C57BL/6J female mice were purchased from The Jackson Laboratory (Bar Harbor, ME, USA). To induce obesity, wild‐type C57BL/6J female mice (Jackson Lab., Bar Harbor, ME, USA) were assigned to two groups randomly at 8 weeks of age. CT group was provided a control diet containing 10% of energy from fat (D12450H, Research Diets, New Brunswick, NJ, USA), and the MO group was provided a high‐fat diet containing 45% of energy from fat (D12451, Research Diets) ad libitum for 10 weeks. Both groups were then mated with randomly selected C57BL/6J males, and mating was confirmed by the presence of a vaginal plug in the morning. At E11.5 and E13.5, embryos were collected after euthanizing the mice by CO_2_ inhalation and cervical dislocation following AVMA Guidelines (2020). All animal studies were conducted in WSU following IACUC‐approved protocol: 6704.

### Single‐Cell RNA Sequencing

Embryos at the same developmental stage were collected during each of the time points, E11.5, and E13.5, and the developmental stages were confirmed following the Theiler stages 19 and 21 with somite numbers 45–47 and 52–55, respectively. By using a dissection microscope (Leica DFC450C), the head and first 7 somite pairs and internal organs were removed. Embryonic tissues were incubated at 37 °C for 15 min with TryLE Express dissociation reagent (Gibco; Cat# 12 605.010) after mincing finely, and the digesta was filtered through a 40 µm cell strainer. According to 10 × Genomics protocol, libraries were constructed by using Chromium Single Cell 3′ Library & Gel Bead Kit v3 and sequenced in an Illumina NovaSeq 600S4 as described in our previous study.^[^ ^27^ ^]^

### Processing of scRNA‐Seq Data and Differential Gene Expression Analysis

Raw sequence was preprocessed by using Cell Ranger V7.0 and mapped with the mouse mm10 reference transcriptome. R package Seurat V 4.3.0 was used to merge libraries from two embryonic stages, and all cells possessing UMIs >200 000 and <500 genes were removed; cells with >20% mitochondrial genes were removed, including doublets, for further analysis. After global normalization, scaling, and reducing the linear dimensionality, top 35 components were processed using Seurat to visualize in a 2D map via Uniform Manifold Approximation (UMAP). The pseudo‐bulk single cell aggregation technique with edgeR assessed gene expression differences between cell clusters and two groups over all clusters. All readings were aggregated using “Seurat2PB” to create the pseudo‐bulk sample. To determine DEGs between CT and MO groups, we employed quantile‐adjusted conditional maximum likelihood (qCML) with a threshold value of *p* < 0.05 after normalizing aggregated data.

### Gene Ontology and KEGG Pathway Enrichment Analysis

For gene ontology analysis, we used the R package “clusterProfiler” and the “gseGO” function for identifying enriched biological and molecular functions associated with the differentially expressed genes between the CT and MO groups. “ChEA3” was used to identify enriched transcription factors associated with the upregulated genes in myogenic clusters of MO embryos.

### Single‐Cell ATAC Seq

Single nuclei were isolated by following protocol CG000124 from 10x genomics. Briefly, the single‐cell suspension from 13.5 embryos was prepared using a similar protocol for scRNA‐seq as described above and diluted to a concentration of 100 cells uL^−1^. To obtain 2  ×  10^6^ cells, 200 uL of the cell suspension was taken and pelleted by centrifuging at 300 ×g for 5 min at 4 °C. Cells were resuspended in 200uL lysis buffer (10 mm Tris‐HCl pH 7.4, 10 mm NaCl, 3 mm MgCl_2_ and 1x PBS) and incubated on ice for 1 min. The suspended cells were then washed with Nuclei wash buffer (10x Genomics PN‐ CG000124), resolved in resuspension buffer (10x Genomics PN‐ CG000124), and centrifuged at 500xg for 10 min at 4 °C twice. The pellets were resuspended in nuclei wash buffer and resuspension buffer, and isolated nuclei were inspected visually, and the nuclei concentration was assessed and proceeded for library preparation according ×10 Genomics single‐cell ATAC‐Seq protocol and sequenced.

### Processing of scATAC‐Seq Data

Raw scATAC‐seq data were analyzed by Cell Ranger ATAC (V 2.1.0) and aligned with the mouse genome reference (mm10). R package Signac (V1.14.0) was used to process data from the CT and MO groups. Data from both groups were merged by using the “merge” function of Signac and normalized by using Term frequency‐inverse document frequency (TF‐IDF). Dimensionality reduction was conducted by singular value decomposition (SVD), and cells were plotted in non‐linear dimension reduction for visualization by the “RunUmap” function, similar to scRNA seq. Gene activity matrices were created by “GeneActivity” function of Signac and merged with ATAC data set as a new assay. To predict the cell types of scATAC‐seq clusters, we transferred the cell label from our scRNA‐seq data by cross‐modality integration. The differentially accessible peaks between cell types were computed with the “FindMarkers” function in Signac and filtered by logfc.threshold  =  0.2 and *p*‐value level *p < 0.05* were visualized by the default coverage plot function.

### Motif Activity Analysis

Transcription factor activity was analyzed using the positional weight matrix of the motif in the JASPER2020 database and using ChromVAR. Motif information from the JASPER database was merged with the Signac Seurat object and the differential activity of motifs was calculated using the “FindMarekrs” function. An adjusted p‐value level *P < 0.05* was used to filter the top differentially active motifs, and the “FindMotifs” function was used to test their fold enrichment and plotted by the “MotifPlot” function.

### Finding Co‐Accessible Sites

Cicero package was used for identifying CCANs using the scATAC‐seq data of myogenic clusters. The myogenic cell cluster data set extracted from the Signac Seurat object were converted to CellDataSet format and cicero object, respectively. The “run_cicero” function was used to find the CCAN network and merged with the dataset to visualize along with the gene accessibility coverage plot.

### Spatial Transcriptomic Sequencing

For special transcriptomic sequencing, a total of 6 embryos from each of the CT and MO groups were selected, where each embryo was randomly selected from one pregnant mouse. Samples were fixed with 10% formalin and embedded with paraffin following formalin‐fixed paraffin‐embedded tissue (FFPE) protocol. Embedded tissues were sectioned at a thickness of 5 µm and mounted on glass slides, dewaxed and incubated overnight with GeoMx RNA detection probes. MYH3 fluorescent‐tagged antibody were used to identify myotome and locate regions‐of‐interest (ROIs). The photocleaved indexing oligos were then collected in the GeoMx Digital Spatial Profiler (Nanostring, Seattle, WA, USA) by profiling ROIs using region‐specific cleaving. To generate a digital quantification of the RNA expression, cleaved indices were sequenced and quantified using Illumina Next Sequeq 2K (San Diego, CA, USA). The downstream analysis was conducted by GeomxTools 3.10.0 through following similar procedure used in our previous study.^[^ ^4^ ^]^

### Cell Culture

C2C12 (ATCC CRL‐1772) myoblast cells were incubated with a growth medium composed of DMEM with 10% FBS (Gibco # 10.439.001) and 1% penicillin‐streptomycin (Sigma, #P0781). When reached 100% confluency, growth medium was supplemented with 2% horse serum to induce myogenic differentiation. The canonical NF‐kB signaling was activated in the differentiating myoblasts by TNF‐a (5 ng mL^−1^). In addition, 500 µm MET (Metformin; MP Biomedicals, Cat# 151 691) or 10 µm CAPE (Caffeic acid phenethyl ester) were used to inhibit the canonical NF‐kB signaling. After 6 days, the myotubes were visualized by immunohistochemical staining using myosin heavy chain (MHC) antibody (MF‐20, DSHB, Iowa City, IA).

### RelA Overexpression

To overexpress RelA, RelA cFlag pcDNA3 (Addgene, Plasmid#20012) were used in C2C12 cells and GFP plasmid as a negative control for evaluation of transfection efficiency. Plasmid transfection was performed using Lipofectamine 3000 transfection kit (Invitrogen) according to manufacturer's protocol. Briefly, in 24‐well plate when the cell reached to 60–70% confluency, 500 ng of plasmid DNA for each well were mixed with P3000 reagent in Opti‐MEM reduced serum media and diluted with equal amount of lipofectamine 3000. The DNA‐lipid complex was incubated for 15 min in RT and added to cells for transfection. The transfection media were replaced with regular growth medium after 24 h of incubation and the transfection efficiency were evaluated based on the expression of GFP.

### RelA Knockdown

For RelA knockdown in C2C12 myoblast cells, we used RelA Pre‐designed Silencer Selected SiRNA (ID: s72857, Invitrogen, CA, USA) and a non‐targeting negative control siRNA. Briefly, 5 nmol of oligo siRNA was diluted to a final concentration of 20 µm. C2C12 cells in the second day of incubation were incubated with a Lipofectamine 3000 Transfection Reagent (Invitrogen) and siRNA (25 nm) in OPTI‐MEM Reduced Serum Medium (Gibco). The transfection medium was replaced by the growth medium after 24 h of incubation.

### RT‐qPCR

Following DNase treatment, total mRNA was extracted from tissue or cells by TRIzol reagent (Sigma, St. Louis, MO), and cDNA was prepared using an iScript cDNA Synthesis Kit (Cat# 1708841, BIO‐RAD) by reverse transcription. The RT‐PCR was conducted using SsoAdvanced Universal SYBR Green Supermix (Bio‐Rad, Cat# 172570) by iQ5 RT‐PCR detection instrument (Bio‐Rad Laboratories, Hercules, CA). The relative expression of mRNA was determined using the ΔΔ− Ct method after normalization to the 18S RNA. Primer sequences used in this study are listed in Table S6 (Supporting Information).

### Western Blotting

Western blot analyses were used to analyze the protein content. Proteins from homogenized tissues and cells were extracted using lysis buffer (100 mm Tris–HCl, pH 6.8, 2.0% SDS, 20% glycerol, 0.02% bromophenol blue, 5% 2‐mercaptoethanol, 100 mm NaF and 1 mm Na3VO4), and separated by 10% sodium dodecyl sulfate‐polyacrylamide gels electrophoresis (SDS‐PAGE) as described previously.^[^ ^27^ ^]^ Antibodies used for western Blotting are listed in the key resources table.

### ChIP‐qPCR Assay

For ChIP‐qPCR, sample fixation, chromatin isolation, immunoprecipitation, and DNA isolation were done by using SimpleChIP Enzymatic Chromatin IP Kit (Cell Signaling Technology, #9003) following the manufacturer's protocol. Chromatin was fragmented through sonication, using 6 × 10s impulses with 1 min pauses. RelA (NF‐κB p65; PCRP‐RELA‐2B6‐s, DSHB, Iowa City, IA) antibody was used for overnight incubation, and spin‐column purification method was used for DNA purification. The purified DNA was then used as a template for qPCR to assess the enrichment of specific target regions. Primers used to amplify the *Myog* promoter and enhancer regions in ChIP‐qPCR are listed in the Table S7 (Supporting Information).

### Statistical Analysis

A total of 15 randomly selected embryos from three pregnant mice for the CT and MO groups were collected respectively for single‐cell RNA sequencing at E11.5 and E13.5 time points and single‐cell ATAC sequencing at E13.5. The transformation, normalization, quality control method and software used for pre‐processing of sequencing data are described above in the “Processing of scRNA‐seq data” and “Processing of scATAC‐seq data” sections. On the other hand, a total of five pregnant mice from each of the CT, MO, and MO‐MET groups were used to collect embryos at E 11.5 and E13.5 for biochemical analysis. Each pregnancy was considered as an experimental unit. For cell culture studies, three separate experiments (*n* = 3) were carried out with 3 technical replicates for each experiment. Data were analyzed using R version 4.3.1, and student's t‐test or one‐way ANOVA was used to compare statistical significance between or among groups. Data are reported as mean ± SEM and visualized using GraphPad Prism version 8.0.0 for Windows (GraphPad Software, Boston, Massachusetts, USA). Statistical differences were indicated as ^∗^ *p* < 0.05, ^∗∗^ *p* < 0.01, and ^∗∗∗^ *p* < 0.001.

## Conflict of Interest

The authors declare no conflict of interest.

## Author Contributions

M.N.H., M.D. designed the study methodology, M.N.H., Y.G., S.I., L.C., X.L., Z.K, and J.M.D.A. conducted the experiments M.N.H., and N.L. analyzed the sequencing data N.L and M. Z. provided key experimental resources. M.N.H. prepared the original draft and M.D. reviewed & edited with input from all the other authors.

## Supporting information



Supporting Information

Supporting Information

## Data Availability

The data that support the findings of this study are openly available in Gene Expression Omnibus (GEO) at https://www.ncbi.nlm.nih.gov/geo, reference number GSE278631.
