# Supplementary material for: RelA Inhibits Embryonic Myogenesis by Coordinately Regulating a Novel Distal Enhancer of Myogenin
Source: Adv Sci (Weinh). 2025 Jul 28;12(38):e03712. doi: 10.1002/advs.202503712 (PMC12520497; doi:10.1002/advs.202503712)
Supplement: Supplementary file 1 — Supporting Information [file ADVS-12-e03712-s001.pdf]

ADVANCED SCIENCE Open Access

Supporting Information

for *Adv. Sci.*, DOI 10.1002/advs.202503712

RelA Inhibits Embryonic Myogenesis by Coordinately Regulating a Novel Distal Enhancer of Myogenin

*Md Nazmul Hossain, Yao Gao, Sharmeen Islam, Li-Wei Chen, Xinrui Li, Zhongyun Kou, Nathan C Law, Jeanene Marie de Avila, Mei-Jun Zhu and Min Du**

Supporting Information

Rela Inhibits Embryonic Myogenesis by Coordinately Regulating a Novel Distal Enhancer of Myogenin

Md Nazmul Hossain, Yao Gao, Sharmeen Islam, Li-Wei Chen, Xinrui Li, Zhongyun Kou, Nathan Law, Jeanene Marie de Avila, Mei-Jun Zhu, and Min Du*

Corresponding author: **Min Du.

Email: min.du@wsu.edu

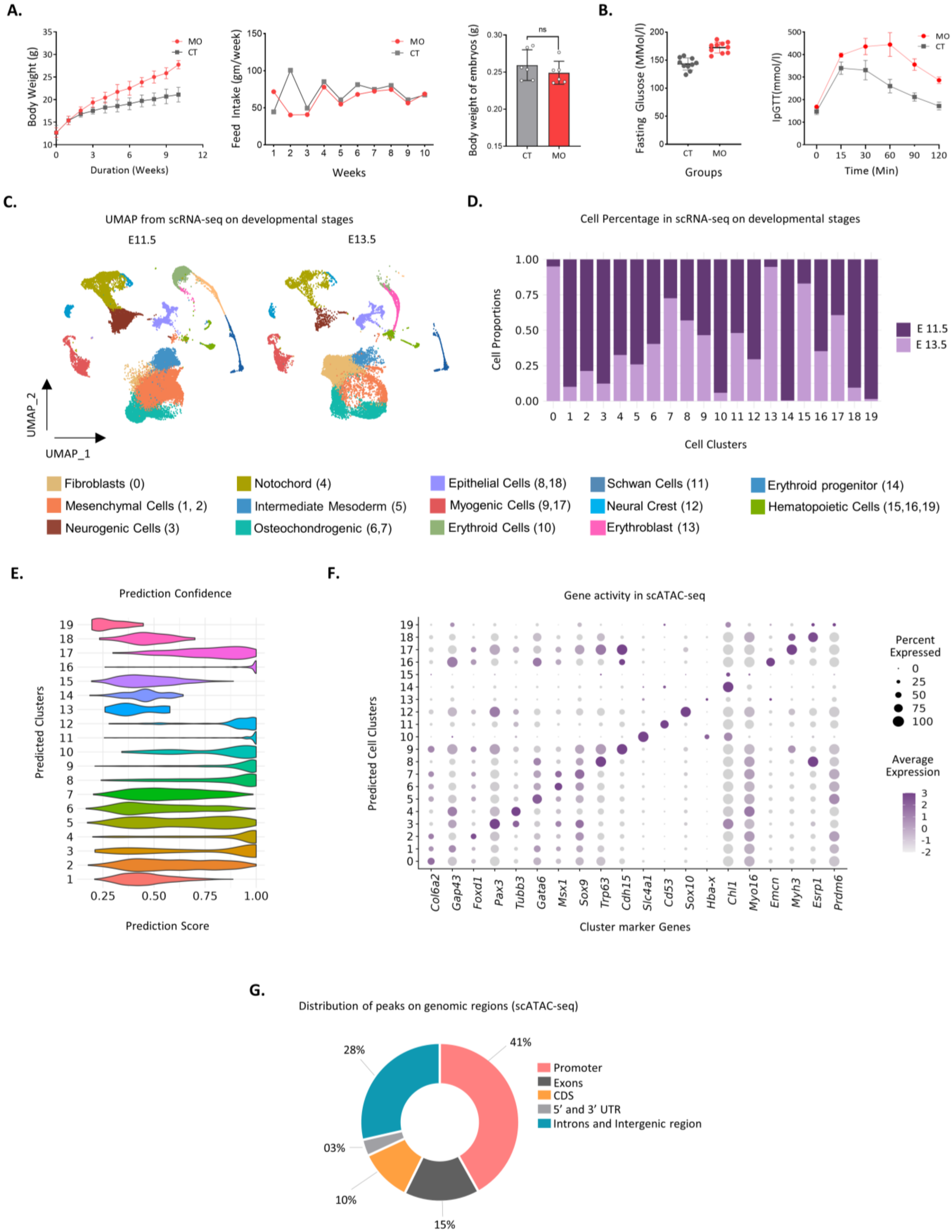

A.
Body Weight (g)
Duration (Weeks)
MO
CT
Feed Intake (gm/week)
Weeks
Body weight of embryos (g)
ns
CT
MO
B.
Fasting Glusose (MMol/l)
Groups
IpGTT(mmol/l)
Time (Min)
C.
UMAP from scRNA-seq on developmental stages
E11.5
E13.5
UMAP_2
UMAP_1
D.
Cell Percentage in scRNA-seq on developmental stages
Cell Proportions
Cell Clusters
E 11.5
E 13.5
Fibroblasts (0)
Mesenchymal Cells (1, 2)
Neurogenic Cells (3)
Notochord (4)
Intermediate Mesoderm (5)
Osteochondrogenic (6,7)
Epithelial Cells (8,18)
Myogenic Cells (9,17)
Erythroid Cells (10)
Schwan Cells (11)
Neural Crest (12)
Erythroblast (13)
Erythroid progenitor (14)
Hematopoietic Cells (15,16,19)
E.
Prediction Confidence
Predicted Clusters
Prediction Score
F.
Gene activity in scATAC-seq
Predicted Cell Clusters
Cluster marker Genes
Col6a2
Gap43
Foxd1
Pax3
Tubb3
Gata6
Msx1
Sox9
Trp63
Cdh15
Slc4a1
Cd53
Sox10
Hba-x
Chl1
Myo16
Emcn
Myh3
Esrp1
Prdm6
Percent Expressed
Average Expression
G.
Distribution of peaks on genomic regions (scATAC-seq)
41%
28%
03%
10%
15%
Promoter
Exons
CDS
5' and 3' UTR
Introns and Intergenic region

Figure S1. Single-cell RNA and ATAC-seq of embryos from control (CT) and obese (MO) mice. (A) Body weight changes of female mice and weight of embryos from CT (control) and MO (maternal obesity) groups (n=10 per group). (B) Fasting blood glucose levels and glucose tolerance test (GTT) in female mice before mating following overnight fasting (n=10 per group). (C) Uniform manifold approximation and projection (UMAP) plot showing 20 major clusters from the scRNA-seq data of E11.5 and E13.5 embryos. (D) Percentage of each cell cluster in the integrated scRNA-seq data of E11.5 and E13.5 embryos. Names of individual clusters are described at the figure along with their corresponding identification numbers. (E) Violin plot shows the prediction confidence of identified cell clusters of scATAC-seq. (F) Dot plot showing top lineage-specific marker gene activity across all the clusters identified from the integrated scATAC-seq. (G) Pie chart showing the distribution of identified peaks from scATAC-seq data in different genomic regions.

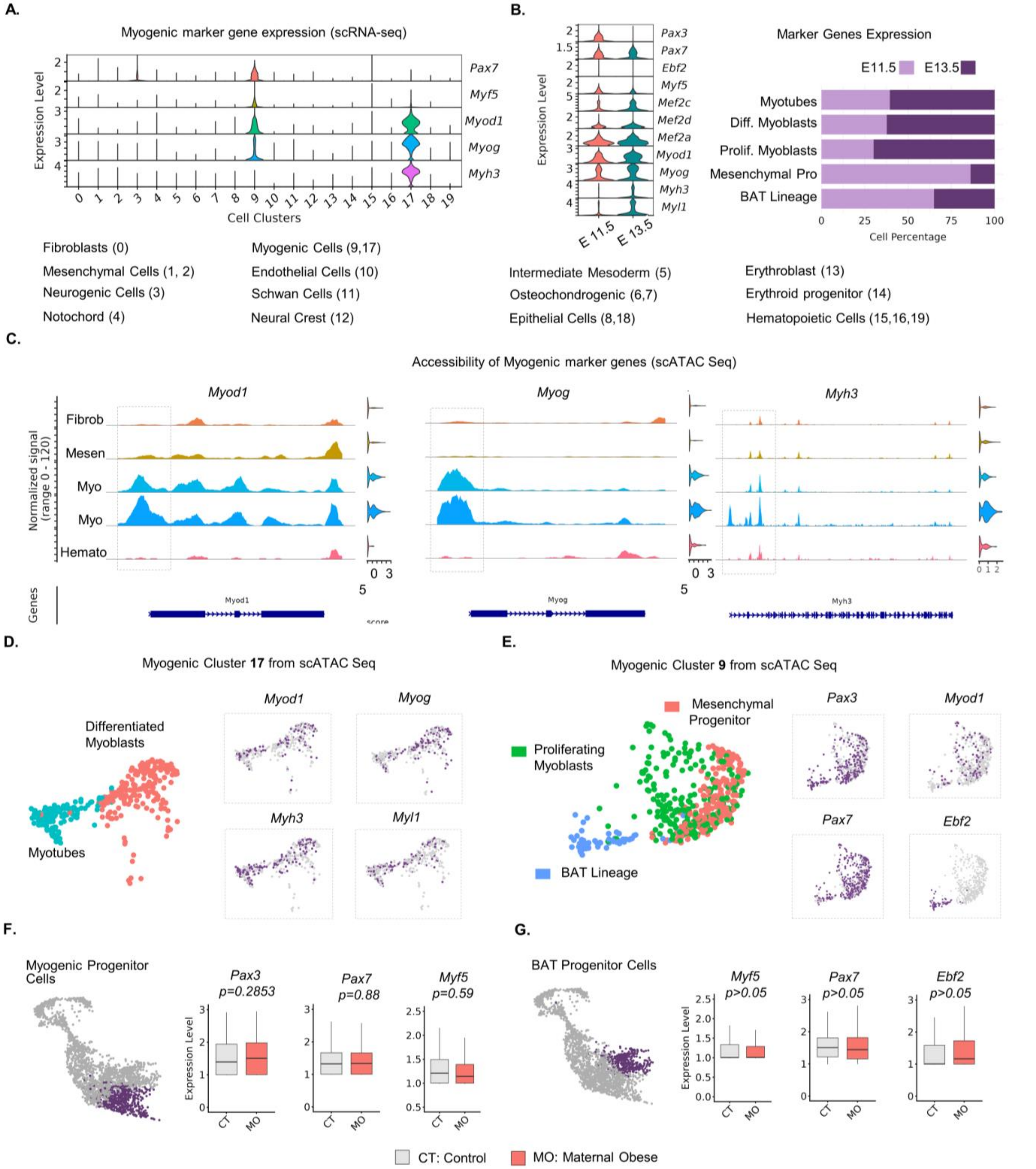

Figure S2. Identification of myogenic cell clusters and reconstruction of myogenic developmental trajectory. (A) Myogenic marker gene expression across all identified cell clusters in scRNA-seq. (B) Myogenic marker gene expression and proportion of different myogenic cell types in myogenic clusters from scRNA-seq of E11.5 and E13.5 embryos. (C) Accessibility of myogenic marker genes in different

cell clusters from scATAC-seq of E13.5 embryos. (D & E) Myogenic marker gene activity in myogenic cell clusters identified from scATAC-seq. Relative expression of cell-type-specific marker genes and myogenic regulatory factor genes in (F) Myogenic Progenitor cells and (G) Brown adipogenic progenitor (BAT) cells from scRNA-seq of E11.5 and E13.5 embryos. CT: Control, MO: Maternal obese.

Fig. S3.

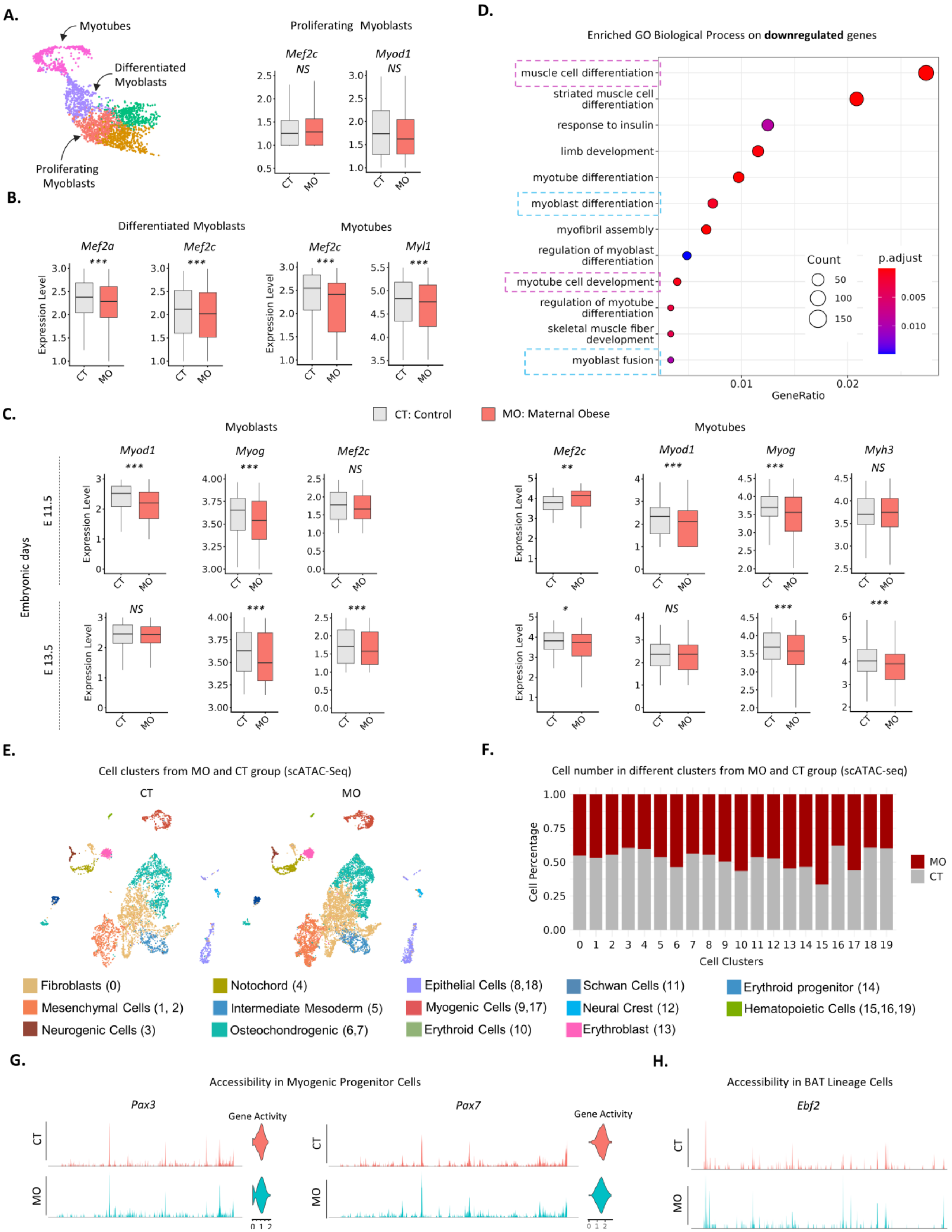
A.
Myotubes
Differentiated Myoblasts
Proliferating Myoblasts
Proliferating Myoblasts
Mef2c
NS
Myod1
NS
CT
MO
B.
Differentiated Myoblasts
Mef2a
Mef2c
Myotubes
Mef2c
Myl1
Expression Level
D.
Enriched GO Biological Process on **downregulated** genes
muscle cell differentiation
striated muscle cell differentiation
response to insulin
limb development
myotube differentiation
myoblast differentiation
myofibril assembly
regulation of myoblast differentiation
myotube cell development
regulation of myotube differentiation
skeletal muscle fiber development
myoblast fusion
Count
50
100
150
p.adjust
0.005
0.010
GeneRatio
C.
CT: Control
MO: Maternal Obese
Myoblasts
Myotubes
Myod1
Myog
Mef2c
Myh3
Embryonic days
E 11.5
E 13.5
E.
Cell clusters from MO and CT group (scATAC-Seq)
CT
MO
Fibroblasts (0)
Mesenchymal Cells (1, 2)
Neurogenic Cells (3)
Notochord (4)
Intermediate Mesoderm (5)
Osteochondrogenic (6,7)
Epithelial Cells (8,18)
Myogenic Cells (9,17)
Erythroid Cells (10)
Schwan Cells (11)
Neural Crest (12)
Erythroblast (13)
Erythroid progenitor (14)
Hematopoietic Cells (15,16,19)
F.
Cell number in different clusters from MO and CT group (scATAC-seq)
Cell Percentage
Cell Clusters
MO
CT
G.
Accessibility in Myogenic Progenitor Cells
Pax3
Pax7
Gene Activity
H.
Accessibility in BAT Lineage Cells
Ebf2

Figure S3. MO impairs embryonic myogenesis via inhibition of *Myog* expression. Relative expression of myogenic regulatory factors in (A) Proliferating myoblasts. (B) Differentiated myoblasts and myotubes from scRNA-seq. (C) Relative expression of myogenic regulatory factors in Myoblasts and Myotubes between MO and CT during E11.5 and E13.5 of embryonic age. (D) Enriched GO biological processes associated with the down-regulated genes in MO embryos. (E) Comparison between Uniform manifold approximation and projection (UMAP) plot showing major cell types from the scATAC-seq of E13.5 embryos from CT and MO embryos. (F) Percentages of different cell clusters. (G) Comparison between the accessibility of Myogenic marker genes in myogenic progenitor cells. (H) BAT marker gene *Ebf2* in BAT progenitor cells between the CT and MO groups based on scATAC-seq. CT: Control; MO: Maternal obese. $*P < 0.05$, $**P < 0.01$, $***P < 0.001$ and NS: non-significant..

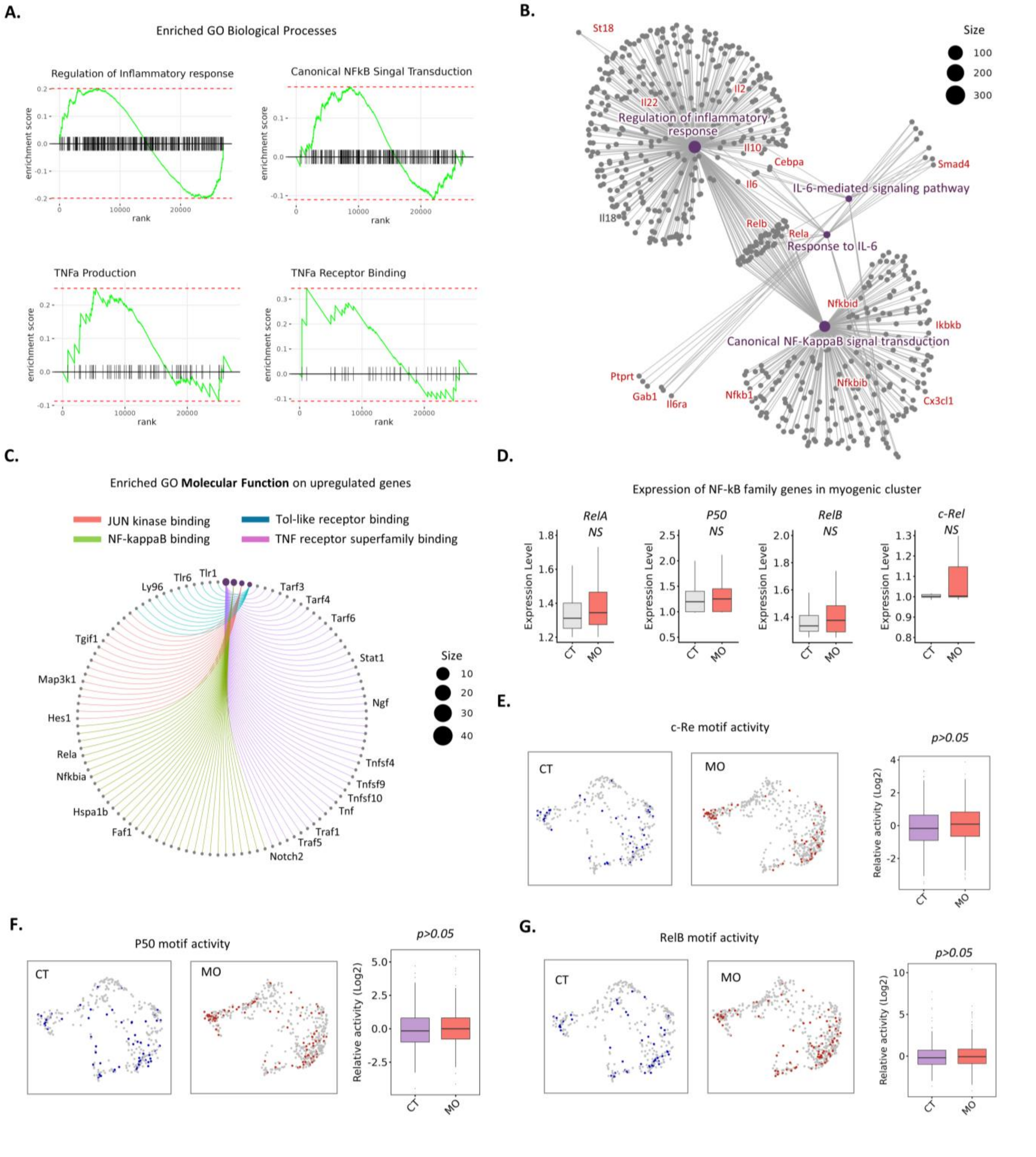

Figure S4. MO induces an inflammatory response and activates NF-kB signaling in embryos. (A) Representative biological processes. (B) Major genes related to inflammatory response identified from GO analysis in upregulated genes in MO compared to CT embryos. (C) Major genes involved in the Enriched GO molecular function associated with the upregulated genes between CT and MO embryos. (D) Relative expression of NF-kB family members in myogenic clusters between MO and CT embryos from scRNA-seq of E13.5 embryos. (E, F & G) Relative motif activity of NF-kB family members in

myogenic cell clusters based on scATAC-seq data. CT: Control, MO: Maternal Obese. *P>0.05* and NS represent non-significant.

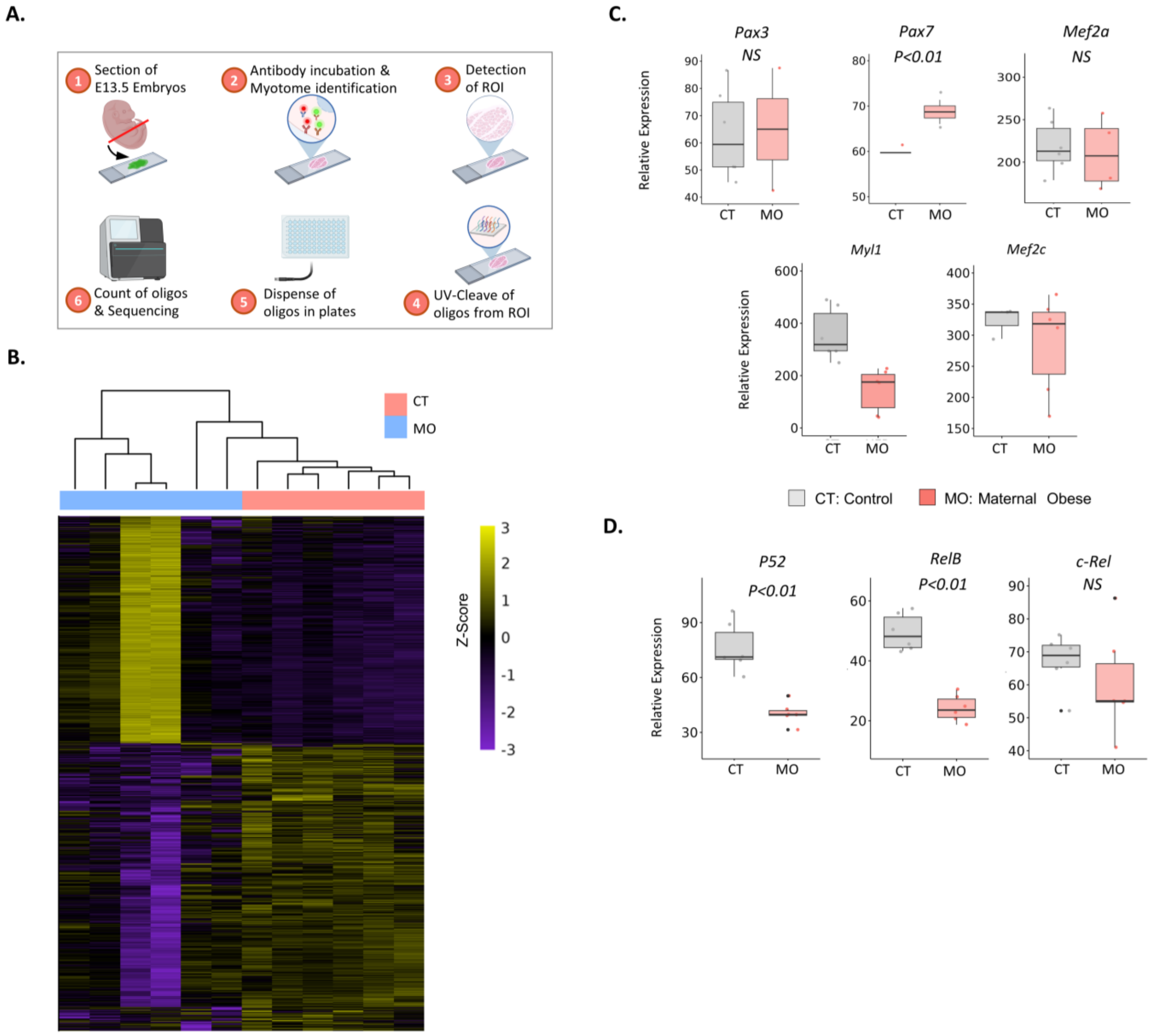

Figure S5. Spatial transcriptomic sequencing of embryonic myotome from CT and MO embryos. (A) Schematic diagram of spatial transcriptomic sequencing of E13.5 embryonic myotome. (B) Heatmap of differential gene expression in embryonic myotome. (C) Relative mRNA abundance of myogenic regulatory factors and marker genes. (D) Members of NF-kB family in the embryonic myotome. CT: Control, MO: Maternal obese. NS: non-significant.

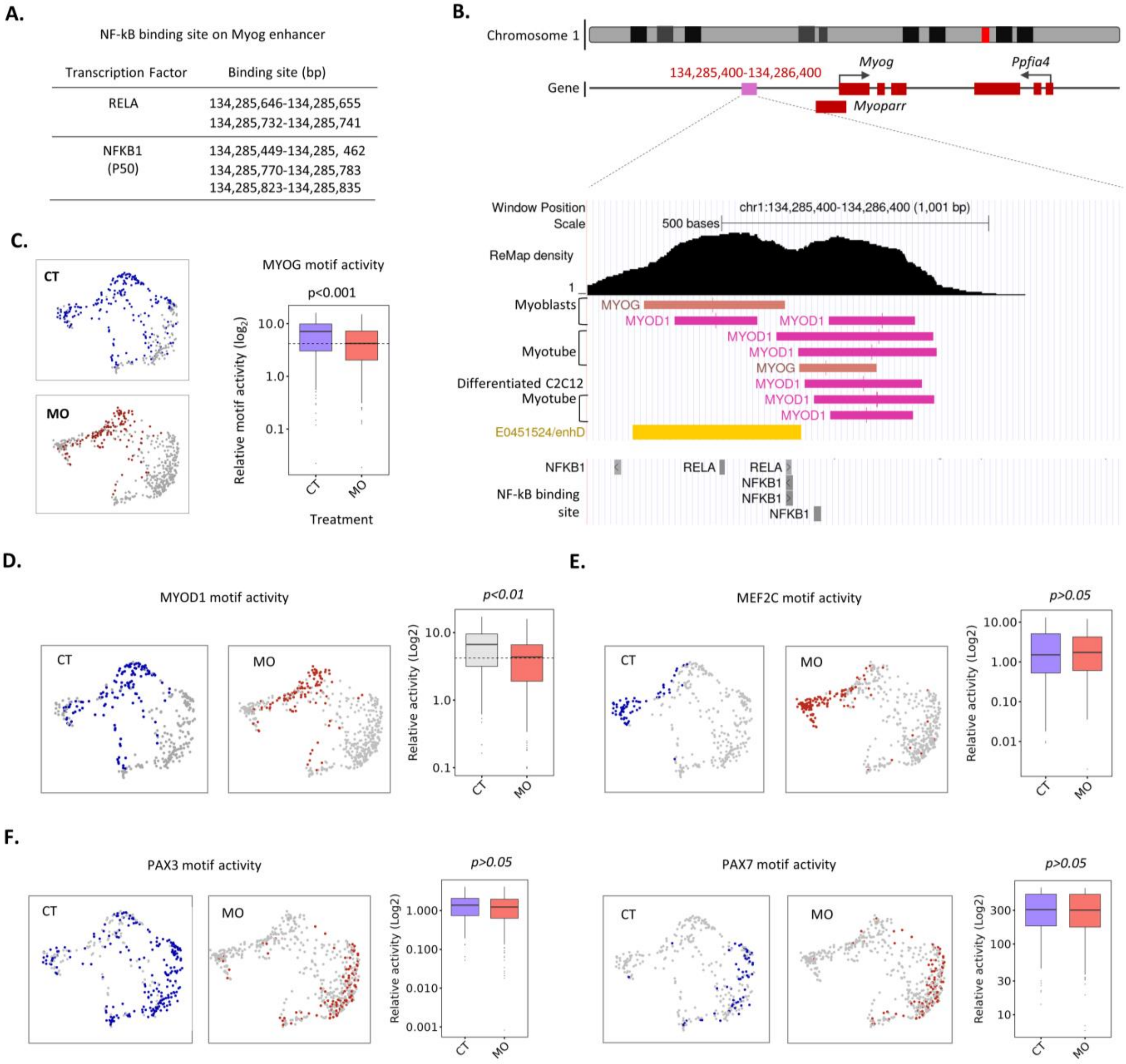

Figure S6. A regulatory enhancer region is involved with regulating *Myog* promoter activity inhibited by NF-kB binding. (A) RELA and P50 motif binding sites on the distal regulatory region (enhancer) of *Myog* gene. (B) MYOG and MYOD1 binding activities in this region in C2C12 cells, embryonic myoblasts and myotubes from ReMap database. (C, D, E & F) Relative motif activity of myogenic marker genes and regulatory factors in myogenic cell clusters from scATAC-seq. CT: Control, MO: Maternal obese. $*P < 0.05$, $**P < 0.01$, $***P < 0.001$, and ns: non-significant.

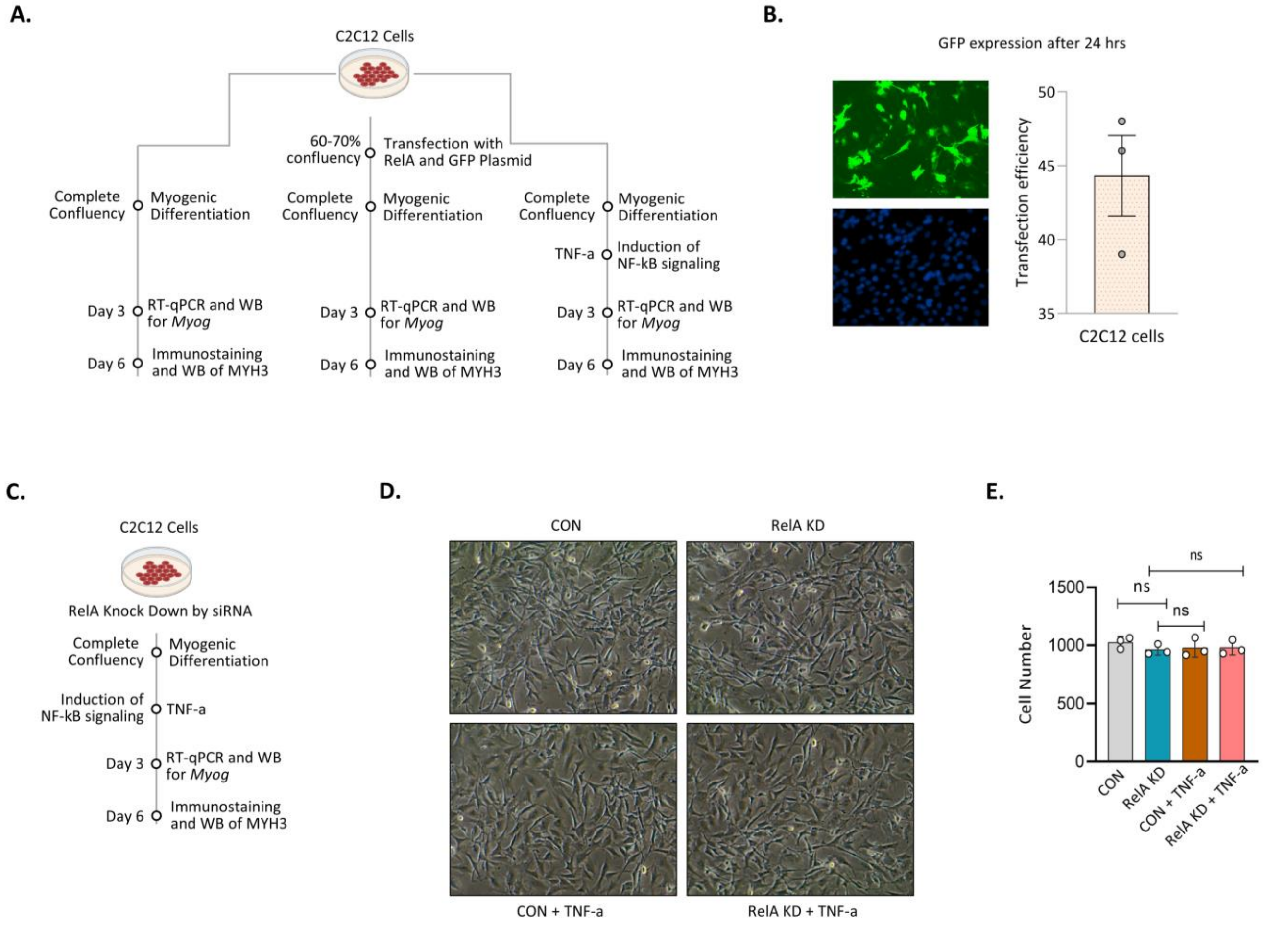

Figure S7. RelA activation inhibits *Myog* and myotube formation in differentiated myoblasts while its inhibition rescues Myogenesis by elevating *Myog* expression. (A) Schematic diagram of RelA overexpression and NF-kB signaling activation by TNF-a in C2C12 myoblast cells. (B) Transfection efficiency after 24 hrs of transfection based on GFP expression (n=3). (C) Schematic diagram of RelA knock down (RelA KD) by siRNA, and NF-kB signaling activation by TNF-a in C2C12 myoblast cells. (D) Bright field image of RelA KD confluent C2C12 cells before initiation of myogenic differentiation. (E) Ratio of differentiated RelA KD myoblasts based on immunofluorescence staining of myotubes with MYH3-antibody. CON: Control. Data are presented as mean ± SEM, and each dot represents one independent experiment. NS: non-significant.

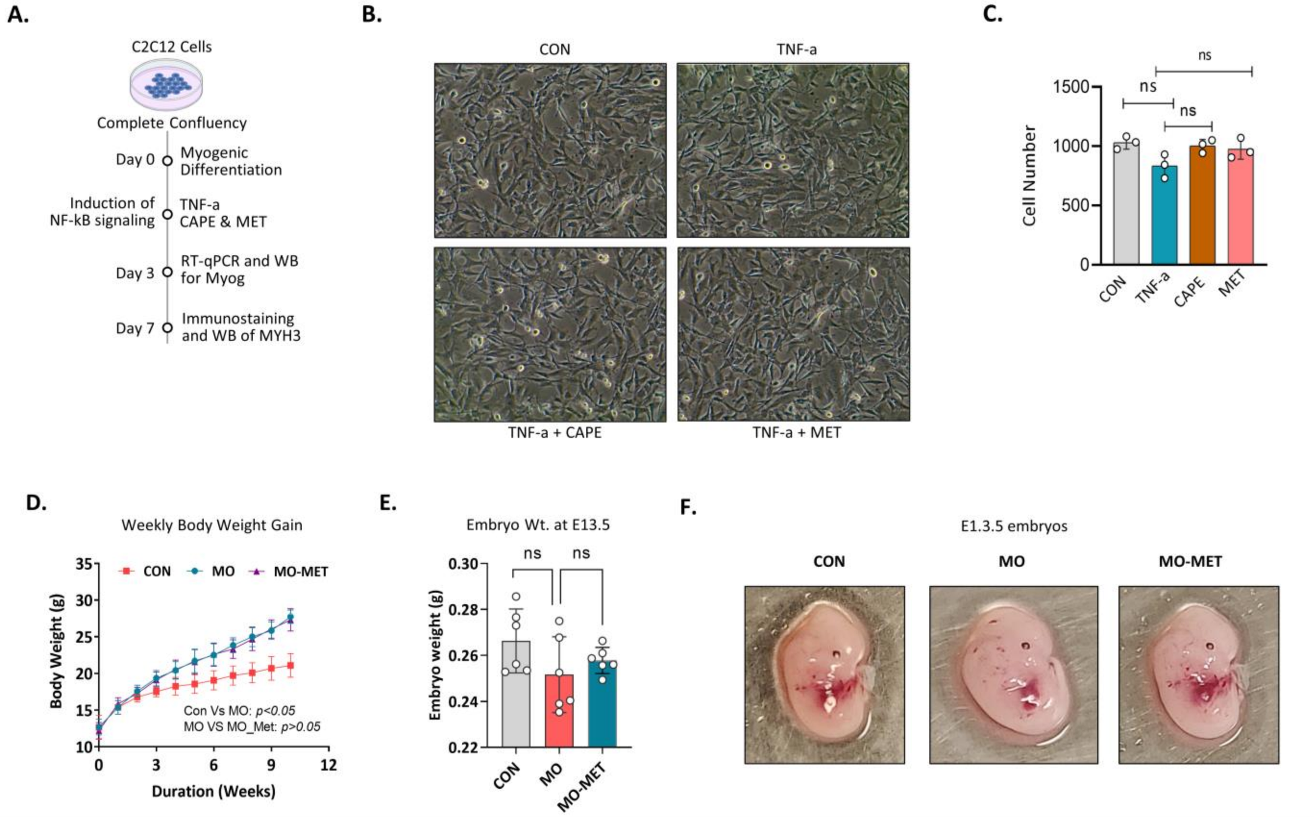

Figure S8. ***In-vivo* treatment of obese mice with metformin attenuates RelA activation and rescues *Myog* expression and embryonic myogenesis in E13.5 embryos.** (A) Schematic diagram of NF-kB signaling activation by TNF-a and its inhibition with CAPE (Caffeic Acid Phenethyl Ester) and MET (Metformin) treatments in C2C12 myoblast cells. (B) Bright field image of confluent C2C12 cells before initiation of myogenic differentiation. (C) Number of differentiated myoblasts based on immunofluorescence staining of myotubes with MYH3 (n=3). (D) Body weight changes of female mice of CT, MO Met groups (n=10 per group). (E) weight of embryos from CON (n = 6 mice per group), MO (n = 6 mice per group) and MO-MET (n = 6 mice per group) groups. (F) Image of embryos from CON, MO, and MO-MET groups from CON: Control, MO: Maternal Obesity, MO-MET: Obese female mouse treated with Metformin. Data are presented as mean ± SEM, and each dot represents one independent experiment. NS: non-significant.
